# NRF2 connects Src tyrosine kinase to ferroptosis resistance in glioblastoma

**DOI:** 10.26508/lsa.202302205

**Published:** 2023-10-25

**Authors:** Claudia Cirotti, Irene Taddei, Claudia Contadini, Claudia Di Girolamo, Gerardo Pepe, Marco De Bardi, Giovanna Borsellino, Manuela Helmer-Citterich, Daniela Barilà

**Affiliations:** 1 Department of Biology, University of Rome “Tor Vergata,” Rome, Italy; 2 Laboratory of Cell Signaling, IRCCS-Fondazione Santa Lucia, Rome, Italy; 3 UOSD Preclinical Models and New Therapeutic Agents Unit, IRCCS Regina Elena National Cancer Institute, Rome, Italy; 4 Neuroimmunology Unit, Experimental Neuroscience, IRCCS Fondazione Santa Lucia, Rome, Italy

## Abstract

Glioblastoma is a severe brain tumor characterized by an extremely poor survival rate of patients. Glioblastoma cancer cells escape to standard therapeutic protocols consisting of a combination of ionizing radiation and temozolomide alkylating drugs that trigger DNA damage by rewiring of signaling pathways. In recent years, the up-regulation of factors that counteract ferroptosis has been highlighted as a major driver of cancer resistance to ionizing radiation, although the molecular connection between the activation of oncogenic signaling and the modulation of ferroptosis has not been clarified yet. Here, we provide the first evidence for a molecular connection between the constitutive activation of tyrosine kinases and resistance to ferroptosis. Src tyrosine kinase, a central hub on which deregulated receptor tyrosine kinase signaling converge in cancer, leads to the stabilization and activation of NRF2 pathway, thus promoting resistance to ionizing radiation-induced ferroptosis. These data suggest that the up-regulation of the Src–NRF2 axis may represent a vulnerability for combined strategies that, by targeting ferroptosis resistance, enhance radiation sensitivity in glioblastoma.

## Abstract (precis)

The hyperactivation of Src tyrosine kinase in glioblastoma leads to the constitutive stabilization and activation of NRF2, thus causing resistance to ionizing radiation-induced ferroptosis.

## Graphical Abstract

**Figure:**
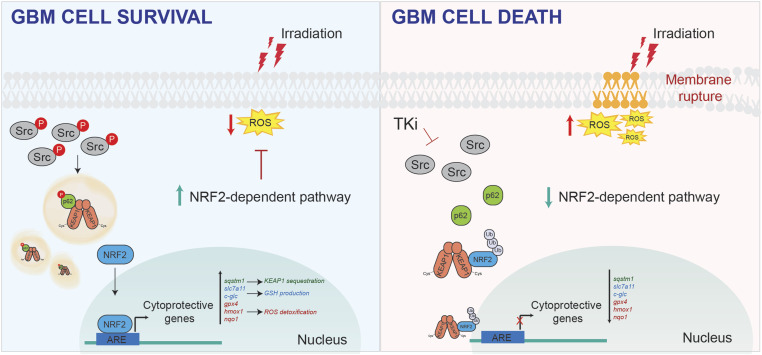

## Introduction

Glioblastoma (GBM) is the most aggressive primary brain tumor in adults characterized by poor prognosis (Ostrom et al, 2015) linked to radiation and chemo therapy resistance. Recent studies reported that failure of radiation therapy is also linked to the ability of cancer cells to counteract ferroptosis (Chen et al, 2021). Ferroptosis is an iron-dependent type of regulated cell death triggered by disproportionate lipid peroxidation, whose alteration is involved both in tumor development and in response to therapy (Chen et al, 2021). The molecular mechanisms that allow cancer cells to overcome ferroptosis have been only partially elucidated.

NRF2 transcription factor, a master regulator of oxidative stress, controls the basal and inducible expression of more than 200 target genes involved in redox homeostasis, metabolism, DNA repair, cell survival, and proliferation (Rojo de la Vega et al, 2018). NRF2 has a dual role in cancer, being both involved in counteracting cancer initiation and in promoting cancer progression and resistance to therapy (Wu et al, 2019). Remarkably, NRF2 up-regulation in cancer can successfully face reactive oxygen species (ROS) generated by radio and chemotherapy, therefore preventing programmed cell death including ferroptosis (Wang et al, 2006; McDonald et al, 2010; Ryoo et al, 2016; Fan et al, 2017; Stockwell et al, 2017). NRF2 counteracts ferroptosis by transactivating several cytoprotective genes involved in iron metabolism, ROS detoxification, and GSH metabolism (Chen et al, 2021). In normal cells, physiological NRF2 turnover, mainly promoted by the KEAP1–CUL3–RBX1 E3–ubiquitin ligase complex controls NRF2 protein levels and its activation. Upon stress conditions, ROS modify reactive cysteine residues on KEAP1, preventing its binding to NRF2, which in turn is stabilized (Itoh et al, 1999; Tebay et al, 2015). Moreover, a noncanonical regulatory pathway involving p62/sequestosome-1 (SQSTM1) protein has been described (Komatsu et al, 2010).

p62 competes with NRF2 for the binding to KEAP1, thus releasing and activating NRF2 (Komatsu et al, 2010; Ichimura et al, 2013). The molecular mechanisms that allow cancer cells to increase NRF2 expression and therefore dampen ferroptosis, have not been fully elucidated and the interplay between NRF2 and other oncogenes deserves further elucidation. Tyrosine kinase (TKs) signaling emerged as a master class of oncogenes (Blume-Jensen & Hunter, 2001; Yang et al, 2022) and represents a core oncogenic requirement for GBM: indeed, up to 50% of GBMs have amplification of a receptor tyrosine kinase (RTK) followed by the aberrant activation of downstream signaling cascade (Snuderl et al, 2011). Src tyrosine kinase is a main node for RTK signaling being both constantly activated by these signals and responsible for the propagation of downstream events, frequently culminating on transcription factor deregulation (Blume-Jensen & Hunter, 2001; Ahluwalia et al, 2010; Cirotti et al, 2020).

Here, we uncover NRF2 as a new molecular player downstream Src deregulation in GBM and we highlight an unrevealed link between the aberrant activation of Src and the inhibition of ionizing radiation (IR)—induced ferroptosis. We also demonstrate that combining Src targeting with ferroptosis induction significantly increases IR sensitivity.

## Results

### Constitutive Src kinase activity promotes NRF2 overexpression in GBM

**Figure 1.**
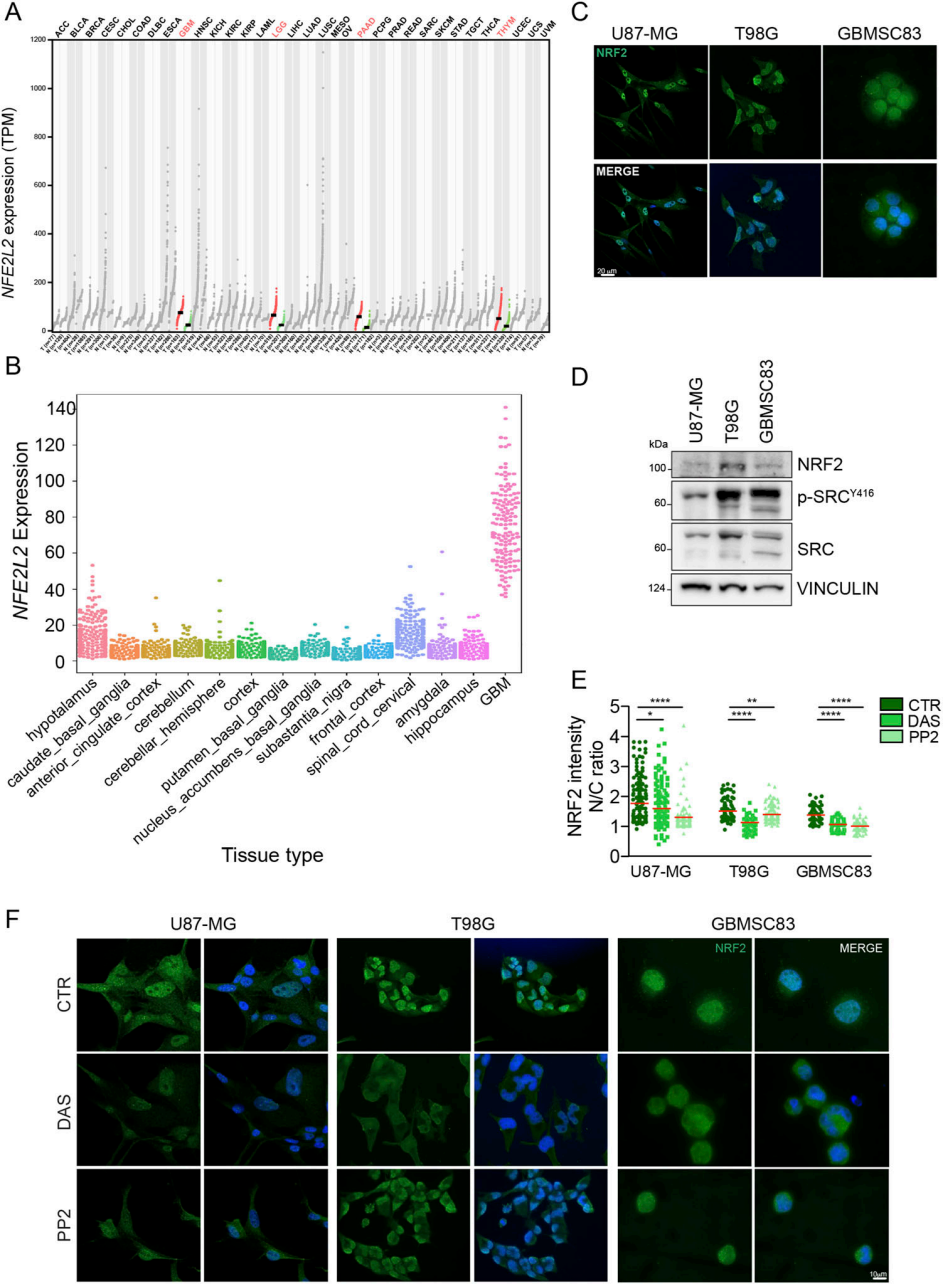
NRF2 is highly expressed in glioblastoma and it is sustained by Src tyrosine kinase activity. **(A)** *NFE2L2* Gene Expression Profile across tumor samples and paired normal tissues. Dot plot; each dot represents *NFE2L2* expression in each sample (ANOVA; match TGCA normal and GTEx data). **(B)** *NFE2L2* gene expression in glioblastoma (The Cancer Genome Atlas–glioblastoma) compared with normal brain areas (GTEx). **(C)** Immunofluorescence of U87-MG, T98G, and GBMSC83 cells. NRF2 (green); DNA (Hoechst, blue). **(D)** Immunoblotting of NRF2, p-Src^Y416^ and Src in U87-MG, T98G, and GBMSC83 cells. Vinculin was used as loading control. **(E, F)** Immunofluorescence experiments of U87-MG, T98G, and GBMSC83 cells upon 16 h treatments with dasatinib 10 nM (U87-MG, T98G) and 1 μM (GBMSC83) or PP2 5 μM. **(E)** Quantification of NRF2 staining reported as the ratio between nuclear and cytosolic fluorescence intensity (N/C). **(F)** Representative images. NRF2 (green); DNA (Hoechst, blue). Results represent the mean of three independent experiments and were analyzed using Mann–Whitney test (*P < 0.05, **P < 0.01, ****P < 0.0001). Source data are available for this figure.

NRF2 transcription factor is aberrantly activated in several types of cancer, sustaining tumor progression, impairing ferroptosis, and leading to resistance to chemo and radiotherapy (Wu et al, 2019). A gene expression profile analysis performed by using GEPIA2 (Gene Expression Profile Interactive Analysis 2) web server (Tang et al, 2019) highlighted increased NRF2 expression in GBM, low-grade glioma, pancreatic adenocarcinoma, and thymoma (Fig 1A). Focusing on GBM, we next compared the expression of NRF2 gene between tumor samples from the Cancer Genome Atlas (TCGA) and normal brain tissues, and we observed a significant increased expression of NRF2 in tumors (Fig 1B). We next moved to in vitro GBM cellular models taking advantage of U87-MG and T98G cell lines and of GBMSC83 neurospheres derived from primary GBM tumors (Mao et al, 2013; Minata et al, 2019; Contadini et al, 2023).

Immunofluorescence analyses confirmed that NRF2 is abundantly expressed and localized in the nuclear compartment in all the GBM cellular models (Fig 1C). Tyrosine kinase deregulation is among the most studied pro-tumoral factor in GBM and Src tyrosine kinase in this tumor is constantly activated (Bjorge et al, 2000; Du et al, 2009). Immunoblotting with anti-pSrc^Y416^ antibody confirmed the constitutive activation of Src in our GBM models (Fig 1D). Given the simultaneous aberrant activation of Src kinase and the high levels of NRF2, we tested whether the pharmacological targeting of Src kinase activity with dasatinib (DAS) and PP2 may affect NRF2 expression.

**Figure S1.**
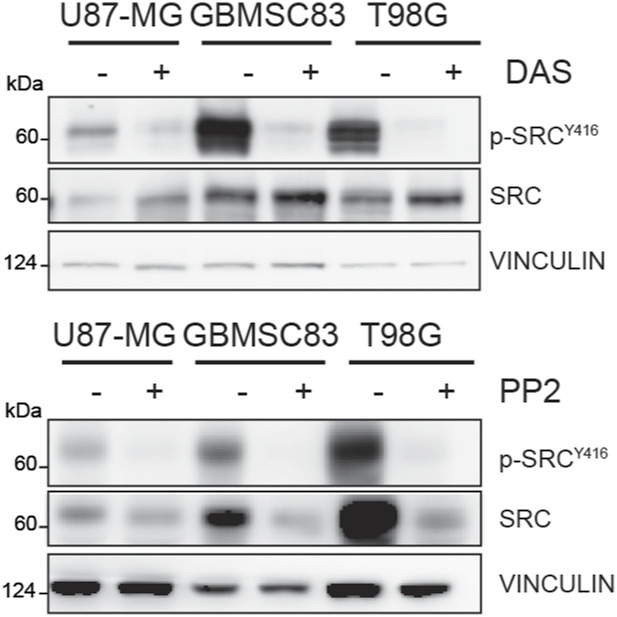
Immunoblotting of p-Src^Y416^ and Src in U87-MG, T98G, and GBMSC83 cells treated with dasatinib 10 nM (U87-MG and T98G) and 1 μM (GBMSC83) (left) or PP2 5 μM (right) for 24 h. Vinculin was used as loading control. Source data are available for this figure.

**Figure S2.**
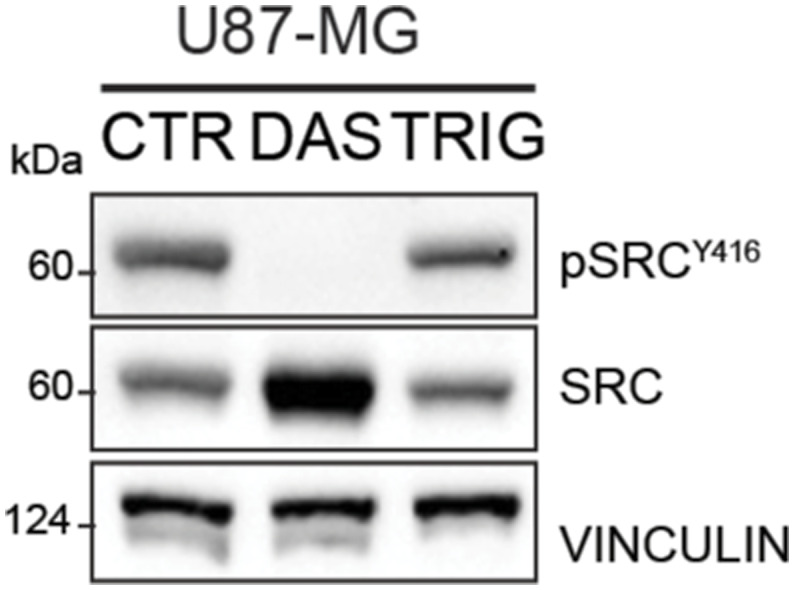
Immunoblotting of p-Src^Y416^ and Src in U87-MG cells treated with dasatinib 10 nM or trigonelline 5 μM for 16 h. Vinculin was used as loading control. Source data are available for this figure.

**Figure 2.**
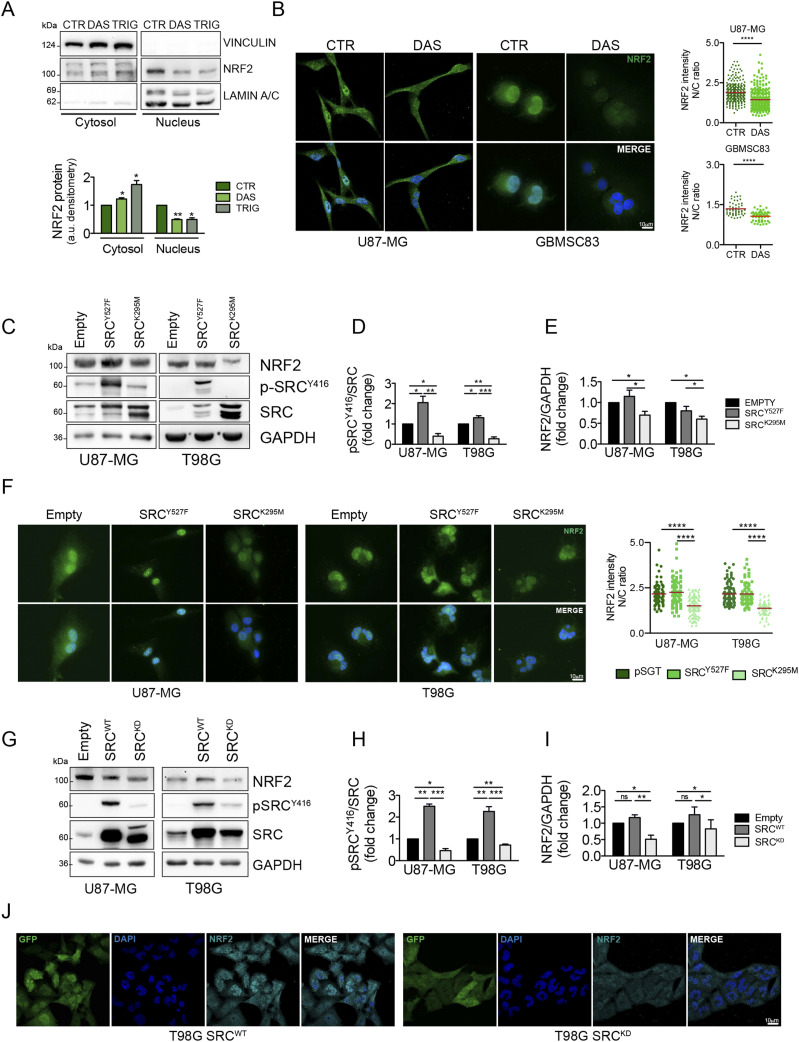
Pharmacological and genetic modulations of Src activity affect NRF2 expression. **(A)** Immunoblotting and relative densitometric analysis of NRF2 in U87-MG cytosolic and nuclear cellular fractions after dasatinib 10 nM or trigonelline (Trig) 5 μM treatment for 6 h. **(B)** Immunofluorescence experiments and relative quantifications of NRF2 staining reported as the ratio between nuclear and cytosolic fluorescence intensities (N/C) in U87-MG and GBMSC83 cells treated for 16 h with dasatinib 10 nM and 1 μM, respectively. NRF2 (green) and DNA (Hoechst, blue). **(C)** Immunoblotting of p-Src^Y416^, Src, and NRF2 in U87-MG and T98G cells transiently transfected for 24 h with empty vector, active Src (Src^Y527F^) or catalytically inactive mutant (Src^K295M^). GAPDH was used as loading control. **(D)** Densitometric analysis of p-Src^Y416^ levels normalized on total Src. **(E)** Densitometric analysis of NRF2 normalized on GAPDH. **(F)** Immunofluorescence and relative quantification of NRF2 staining in the same cells. NRF2 (green); DNA (Hoechst, blue). **(G)** Immunoblotting of p-Src^Y416^, Src, and NRF2 in U87-MG and T98G stably overexpressing the empty vector, *wild-type* Src (Src^WT^) or the catalytically inactive mutant (Src^K295M^, hereafter Src^KD^). GAPDH was used as loading control. **(H)** Densitometric analysis of p-Src^Y416^ levels normalized on total Src. **(I)** Densitometric analysis of NRF2 normalized on GAPDH. **(J)** Immunofluorescence of T98G Src^WT^ and Src^KD^ cells. NRF2 (cyan); GFP (green); DNA (Hoechst, blue). Results represent the mean of at least three independent experiments (±SEM). **(A, D, E, H, I)** Statistical analyses: paired *t* test (A, D, E, H, I) (*P < 0.05; **P < 0.01; ***P < 0.001). **(B, F)** Mann–Whitney test or unpaired *t* test according to normal distribution (B) and ANOVA test followed by Kruskal–Wallis test (F) (****P < 0.001). Source data are available for this figure.

Interestingly, immunofluorescence experiments show that the inhibition of Src (Fig S1) significantly decreased NRF2 nuclear localization (Fig 1E and F). Furthermore, NRF2 localization was evaluated also upon subcellular fractionation. Pharmacological Src kinase inhibition (Fig S2) significantly impinged on NRF2 nuclear localization, similarly to what was observed when cells were treated with trigonelline, a well-known NRF2 inhibitor (Arlt et al, 2013) (Fig 2A). Confocal microscopy analyses further confirmed that DAS significantly affected NRF2 nuclear localization, resulting in a reduced ratio of nuclear/cytoplasmic NRF2 fluorescence intensity (Fig 2B). To unambiguously demonstrate that Src may modulate NRF2, Src kinase activity was also genetically modulated taking advantage of Src mutants that either mimic the constitutive activation (Src^Y527F^) or the enzymatic inactivation (Src^K295M^). Interestingly, the overexpression of Src^K295M^, resulting in a significant inhibition of Src activity compared with both empty vector and Src^Y527F^ overexpressing cells (Fig 2C and D), caused a decrease of NRF2 protein expression (Fig 2C and E) and nuclear localization (Fig 2F). We next generated GBM cell lines stably overexpressing *wild-type* Src (Src^WT^) or the catalytically inactive mutant Src^K295M^ (hereafter named Src kinase-dead, Src^KD^) in which we could once again recapitulate that Src activity is significantly inhibited after Src^KD^ overexpression (Fig 2G and H) and required to sustain NRF2 (Fig 2G, I, and J). Overall, these data allow the conclusion that Src activity sustains NRF2 expression and nuclear localization in GBM cells.

### Src kinase activity promotes NRF2 signaling

**Figure 3.**
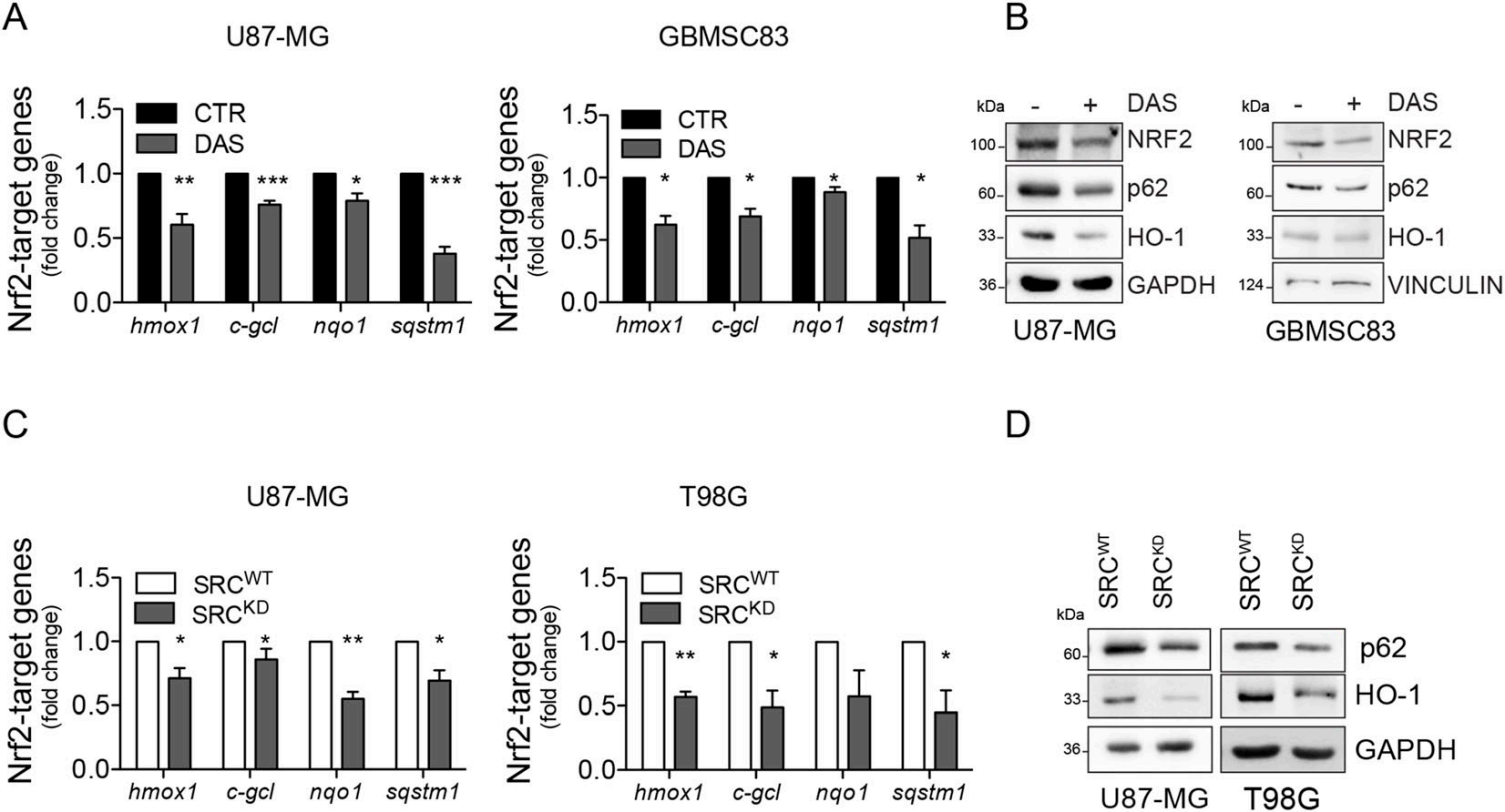
Pharmacological and genetic modulations of Src activity affect NRF2 transcriptional activity. **(A)** Real-time PCR of NRF2 target genes in U87-MG and GBMSC83 cells treated for 16 h with dasatinib 10 nM and 1 μM, respectively (*actin*: housekeeping gene). **(A, B)** Immunoblotting of NRF2, p62, and HO-1 n U87-MG and GBMSC83 cells treated as in (A). GAPDH and vinculin were used as loading controls. **(C)** Real-time PCR of NRF2 target genes in U87-MG and T98G cells stably overexpressing Src^WT^ or Src^KD^ (*actin*: housekeeping gene). **(C, D)** Immunoblotting of p62 and HO-1 in cells as in (C). GAPDH was used as loading control. Results represent the mean of at least three independent experiments (±SEM). Statistical analyses: paired *t* test (*P < 0.05; **P < 0.01; ***P < 0.001). Source data are available for this figure.

**Figure S3.**
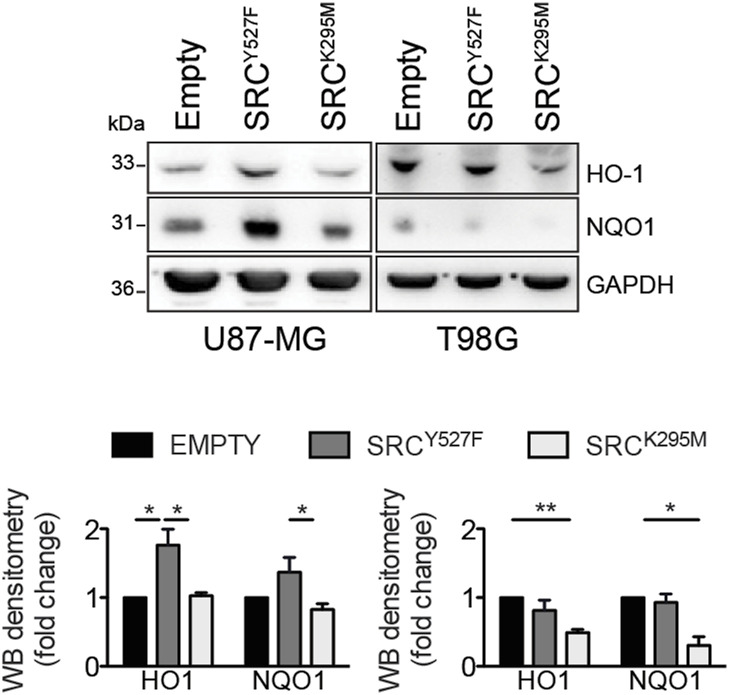
Immunoblotting and relative densitometric analysis of NRF2 targets, HO-1, and NQO1, in U87-MG and T98G cells after 24 h of transient transfection with active Src (Src^Y527F^) or catalytically inactive mutant (Src^K295M^). GAPDH was used as loading control. Source data are available for this figure.

To assess whether Src kinase may modulate NRF2 functionality, NRF2 transcriptional activity was evaluated upon pharmacological or genetic modulation of Src. Remarkably, DAS treatment triggered the down-regulation of several NRF2 target genes, namely *heme oxygenase 1* (*hmox1*), *glutamate-cysteine ligase catalytic subunits* (*c-gcl*), *NAD(P)H Quinone Dehydrogenase 1* (*nqo1*), and *sequestosome-1* (*sqstm1*), as revealed by real-time PCR (RT–qPCR), both in U87-MG cells and in GBMSC83 neurospheres (Fig 3A). Consistently immunoblotting analyses revealed a significant decrement of SQSTM1/p62 (hereafter, p62) and heme oxygenase 1 (HO-1) proteins upon DAS treatment (Fig 3B). The transcriptional activity of NRF2 was also significantly decreased in U87-MG and T98G cells engineered to stably express Src^KD^, compared with the ones expressing Src^WT^ counterpart (Fig 3C and D) and upon transient transfection of the catalytically inactive in Src^K295M^ (Fig S3). Overall, these data suggest that Src activity sustains NRF2 transcriptional activity.

### Src kinase activity inhibition turns off NRF2 pathway affecting p62–KEAP1 interaction

**Figure 4.**
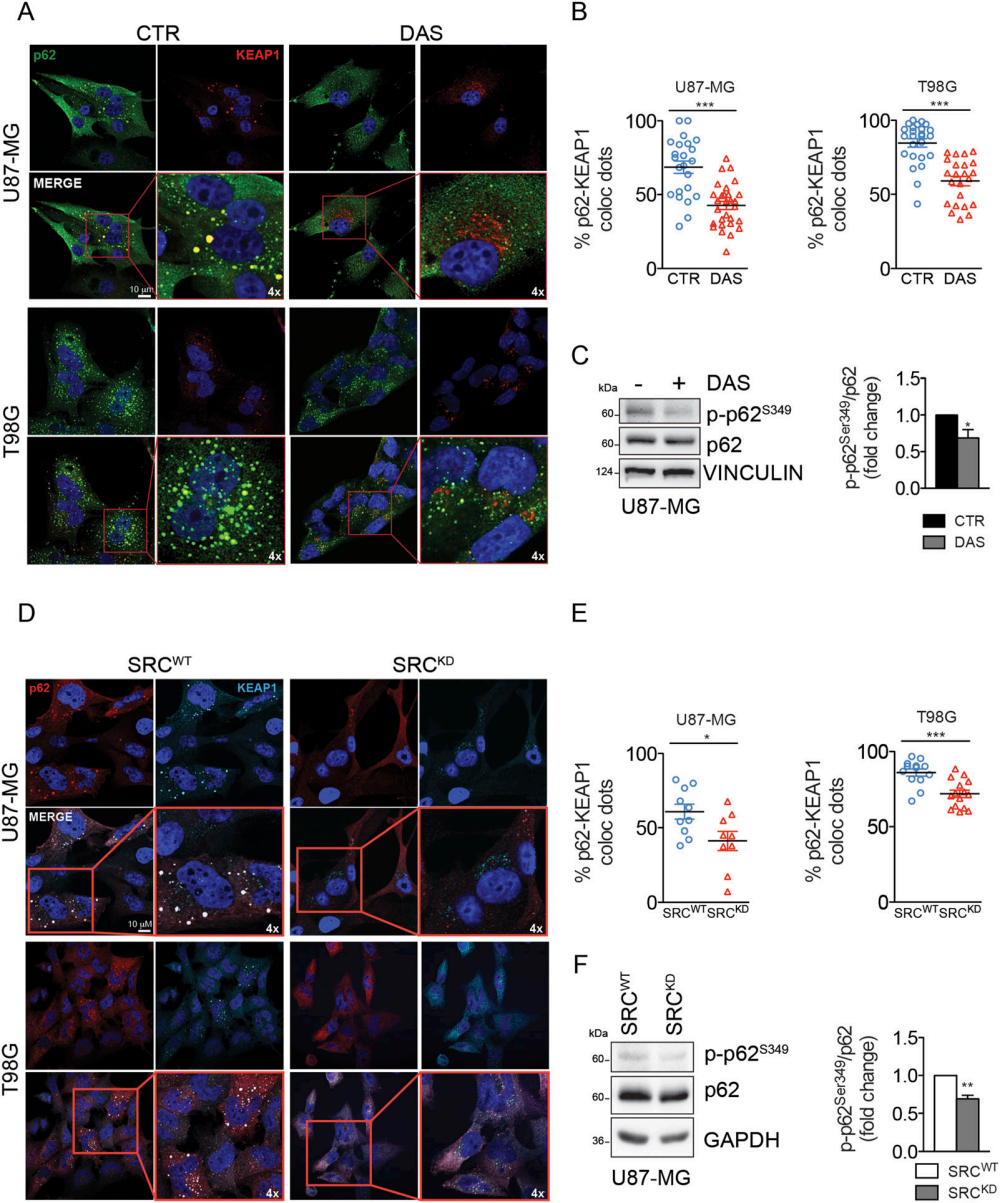
Src activity sustains p62–KEAP1 interaction promoting p62S349 phosphorylation. **(A, B)** Confocal microscopy analyses and (B) quantification of colocalizing dots in U87-MG and T98G cells treated with dasatinib 10 nM for 16 h p62 (green); KEAP1 (red); DNA (Hoechst, blue); 4x digital magnification showing merged signals. **(C)** Immunoblotting and relative densitometric analysis of p-p62^S349^ and p62 in U87-MG cells treated as previously. Vinculin was used as loading control. **(D, E)** Confocal microscopy analyses and (E) quantification of colocalizing dots in U87-MG and T98G cells stably overexpressing Src^WT^ or Src^KD^. p62 (red); KEAP1 (cyan); DNA (Hoechst, blue); 4x digital magnification showing merged signals. **(F)** Immunoblotting and relative densitometric analysis of p-p62^S349^ and p62 in U87-MG cells stably overexpressing Src^WT^ or Src^KD^. GAPDH was used as loading control. Results represent the mean of at least three independent experiments (±SEM). **(B, C, E, F)** Statistical analyses: paired (C, F) or unpaired (B, E) *t* test: (*P < 0.05; **P < 0.01; ***P < 0.001). Source data are available for this figure.

Previous work highlighted a strong correlation between NRF2 expression and p62 in GBM tumors, and demonstrated that the high expression levels of NRF2 protein may be sustained by the ability of p62 to bind KEAP1 preventing its interaction with NRF2 (Pölönen et al, 2019). We therefore asked the question whether the aberrant activity of Src may sustain p62–KEAP1 interaction therefore enhancing NRF2 expression and activity. Confocal microscopy analysis on GBM cells treated or not with DAS, highlighted the presence of p62 aggregates and a strong colocalization between p62 and KEAP1 in these structures (Fig 4A and B). More interestingly, DAS treatment significantly released p62–KEAP1 interaction and promoted a diffused relocalization of KEAP1 into the cytosolic compartment, particularly in the perinuclear area (Fig 4A and B). The competitive interaction between p62 and KEAP1 is ensured by the phosphorylation of p62 on serine 349 residue (S349) (Ichimura et al, 2013). Interestingly, DAS treatment severely impinged on this phosphorylation (Fig 4C), suggesting that Src activity could indirectly promote p62 phosphorylation on S349 which drives the formation of KEAP1–p62 complex, ultimately leading to NRF2 stabilization and accumulation. Confocal microscopy analyses on Src^WT^ or Src^KD^ cells further confirmed this hypothesis (Fig 4D and E). Furthermore, Src^KD^ triggered the reduction of p62 phosphorylation on S349 (Fig 4F), similarly to what observed with DAS. These data demonstrated that Src kinase activity sustains p62–KEAP1 interaction therefore regulating NRF2 functionality.

### Src kinase activity inhibition promotes NRF2–KEAP1 interaction triggering NRF2 degradation

**Figure 5.**
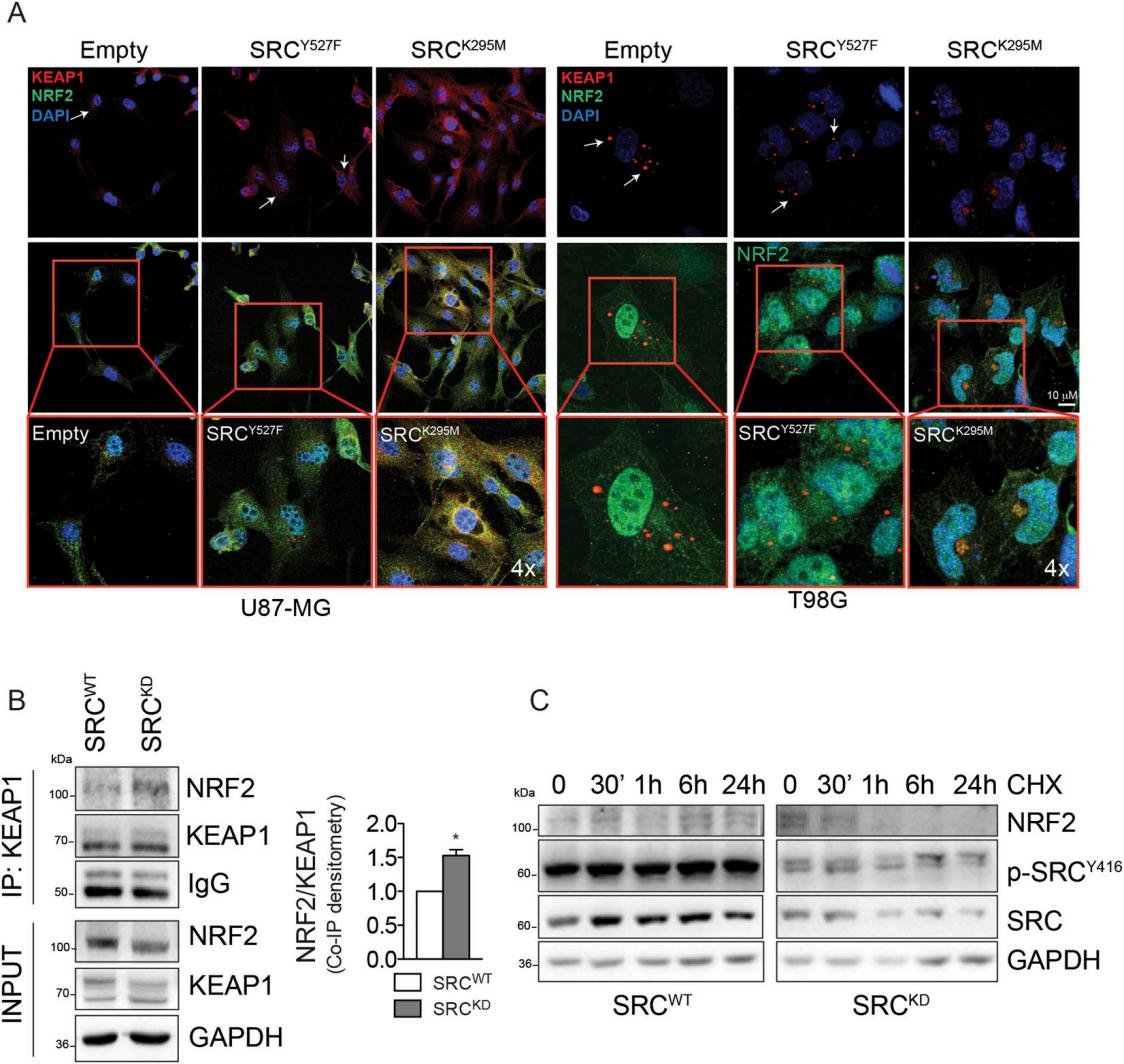
Src activity affects KEAP1 intracellular distribution and its interaction with NRF2. **(A)** Confocal microscopy analyses in U87-MG and T98G cells after 24 h of transient tranfection with active Src (Src^Y527F^) or catalytically inactive mutant (Src^K295M^). NRF2 (green); KEAP1 (red); DNA (Hoechst, blue). 4x digital magnification showing merged signals. **(B)** Co-immunoprecipitation experiments and relative quantification of KEAP1 in U87-MG cells stably overexpressing Src^WT^ or Src^KD^. **(C)** Immunoblotting of NRF2, p-pSrc^Y416^, and Src in U87-MG Src^WT^ and Src^KD^ treated with cycloheximide 100 μg/ml for 0, 30 min, 1, 6, and 24 h. GAPDH was used as loading control. Results represent the mean of three independent experiments (±SEM). Statistical analysis: paired *t* test: (*P < 0.05). Source data are available for this figure.

The observation that the inhibition of Src activity leads to the release of KEAP1–p62 interaction supported the hypothesis that in this context Src inhibition may conversely promote the interaction between KEAP1 and NRF2. Importantly, we could show that Src^K295M^ transient transfection drove the colocalization between KEAP1 and NRF2 (Fig 5A, red boxes—4x magnification). Of note, we immunoprecipitated KEAP1 from U87-MG Src^WT^ and Src^KD^ cells and we observed that the interaction between KEAP1 and NRF2 is significantly increased in Src^KD^ cells, despite of the decreased levels of NRF2 protein in this condition (Fig 5B). Consistently, Src^KD^ overexpression decreased NRF2 protein stability (Fig 5C). Altogether, these data strongly support the conclusion that Src kinase activity enhances NRF2 protein stability.

### Src activity sustains mTORC1-dependent p62 phosphorylation

**Figure 6.**
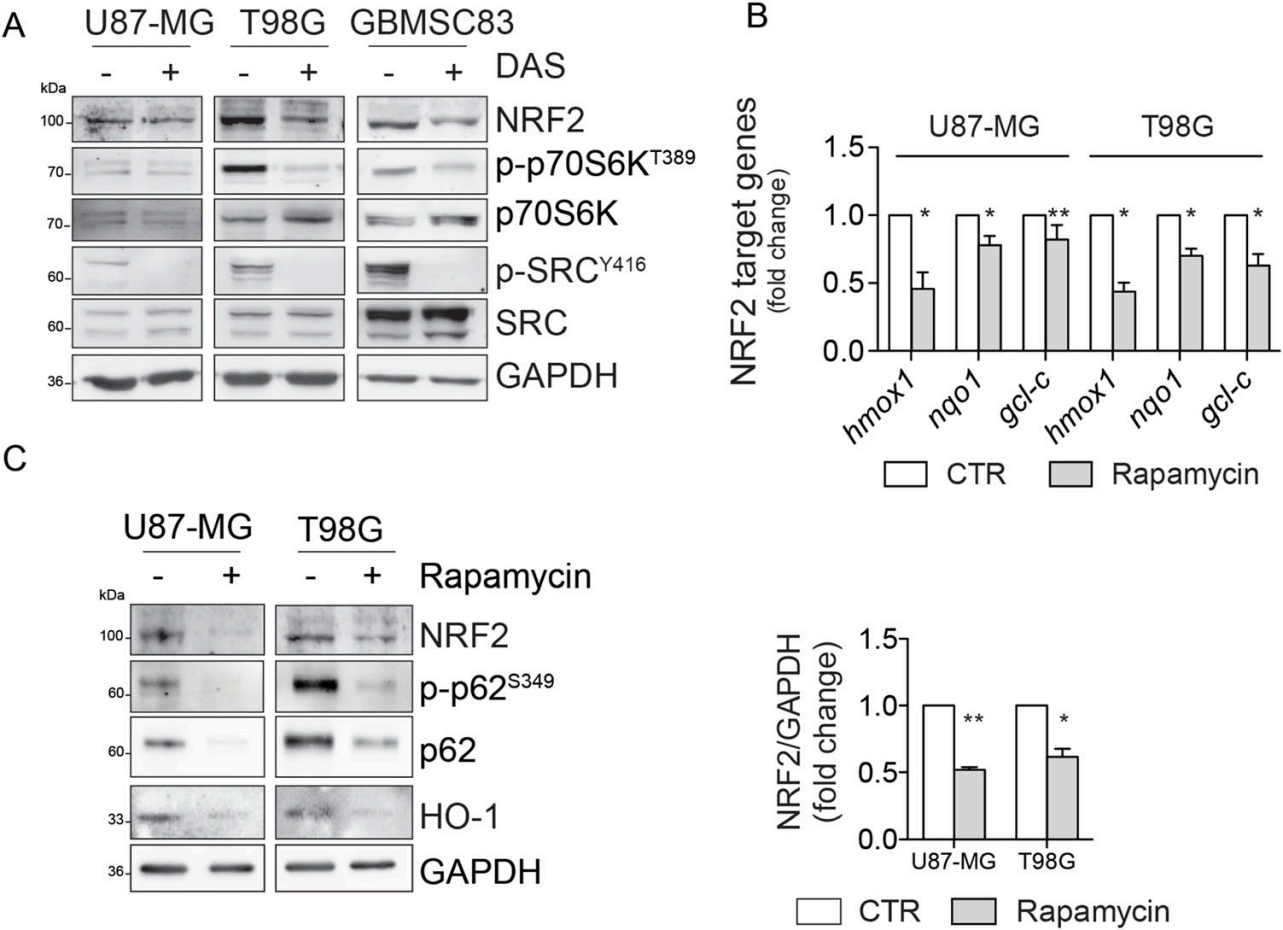
Src activity sustains mTORC1-dependent signaling. **(A)** Immunoblotting of p-p70S6K^T389^ and p70S6K in U87-MG, T98G, and GBMSC83 cells treated for 16 h with dasatinib 10 nM and 1 μM, respectively. GAPDH was used as loading control. **(B)** Real-time PCR of NRF2 target genes in U87-MG and T98G cells treated with rapamycin 100 nM for 48 h (actin: housekeeping gene). **(C)** Immunoblotting of NRF2, p-p62^S349^, p62, and HO-1 in U87-MG, and T98G cells treated as previously, and relative NRF2 densitometric analysis. GAPDH was used as loading control. Results represent the mean of three independent experiments (±SEM). Statistical analyses: paired *t* test: (*P < 0.05; **P < 0.01). Source data are available for this figure.

**Figure S4.**
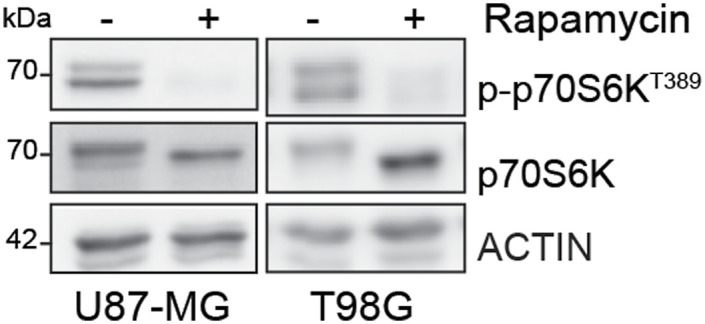
Immunoblotting of p-p70S6K^T389^ and p70S6K in U87-MG and T98G cells treated with rapamycin 100 nM for 48 h. Actin was used as loading control. Source data are available for this figure.

**Figure S5.**
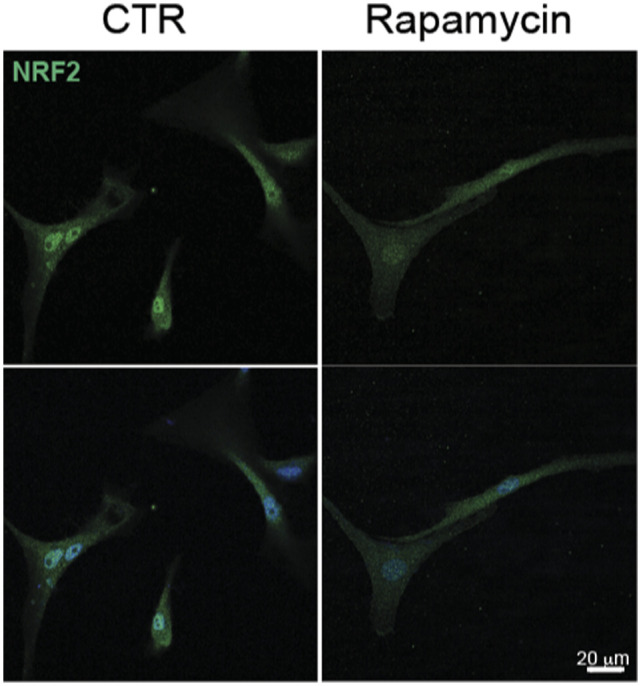
Immunofluorescence staining in U87-MG cells treated with rapamycin 100 nM for 48 h. NRF2 (green); DNA (Hoechst, blue).

**Figure S6.**
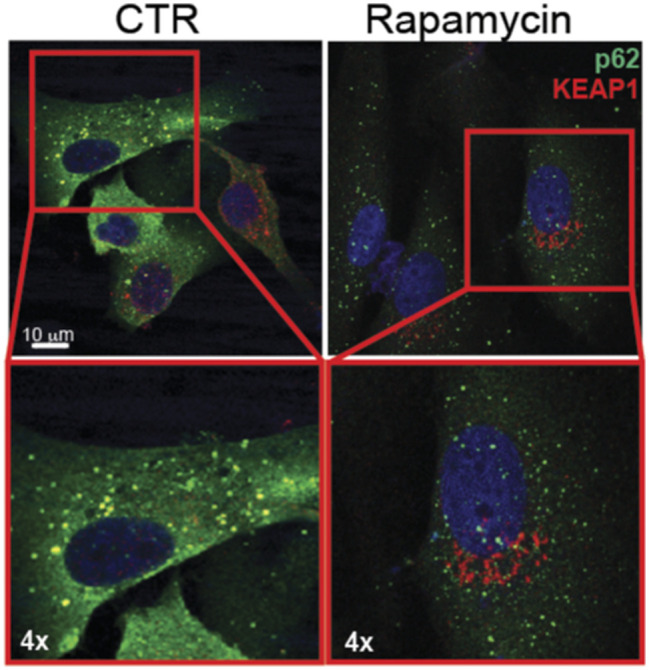
Confocal microscopy analyses of U87-MG cells treated with rapamycin 100 nM for 48 h. p62 (green); KEAP1 (red); DNA (Hoechst, blue); 4x digital magnification showing merged signals.

**Figure S7.**
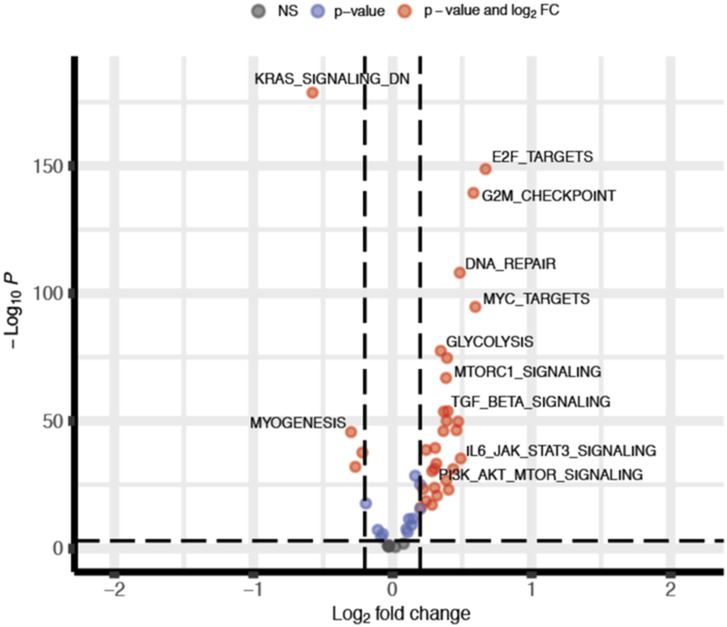
Volcano plot. The log_2_ FC indicates the mean activity level for each pathway. Each dot represents one pathway. Black and blue dots represent pathways with no significant *P*-value and no significant FC, respectively, red dots represent up- and down-regulated pathways in Glioblastoma (the Cancer Genome Atlas data) compared with normal brain cortex (GTEx data).

It has been previously reported that the competitive binding between p62 and KEAP1 proteins is ensured by mTORC1-dependent phosphorylation of p62 on serine 349 residue (Ichimura et al, 2013). Data from literature also support a direct role for Src kinase in modulating mTORC1 activity in several types of cancers (Vojtěchová et al, 2008; Pal et al, 2018), raising the question whether the constitutive activity of Src may trigger the aberrant activation of mTORC1 in GBM. Immunoblotting experiments revealed the presence of phosphorylated p70S6K (p-p70S6K^T389^), a well-known target of mTORC1 (Maiese et al, 2013) in our cellular models (Fig 6A). Remarkably, pharmacological targeting of Src kinase activity resulted in the down-regulation of p70S6K phosphorylation (Fig 6A). To further confirm the involvement of mTORC1 in sustaining Src-dependent NRF2 hyperactivation, we took advantage of the mTORC1 inhibitor rapamycin. As expected, rapamycin turned off mTORC1 activity, as shown by the down-regulation of p-p70S6K^T389^ (Fig S4). Of note, NRF2-dependent pathway was strongly affected by rapamycin, as shown by the decreased expression levels of NRF2 and of its target genes (Figs 6B and C and S5). In addition, rapamycin treatment dampened p62 phosphorylation on S349, thus impairing KEAP1 sequestration (Figs 6C and S6). Overall, these data suggest that Src may depend on mTORC1 activation to sustain p62 phosphorylation and NRF2 hyperactivation in GBM cellular models. Interestingly, comparing the expression profiles of GBM patients (TGCA data) with normal brain tissues (GTEx data) we found a significant up-regulation in tumor samples of several gene-enriched mTORC1 pathways, thus strengthening the relevance of this hallmark in GBM (Fig S7).

### NRF2 is required for Src-dependent proliferation, clonogenicity, and resistance to ionizing radiation treatment

**Figure 7.**
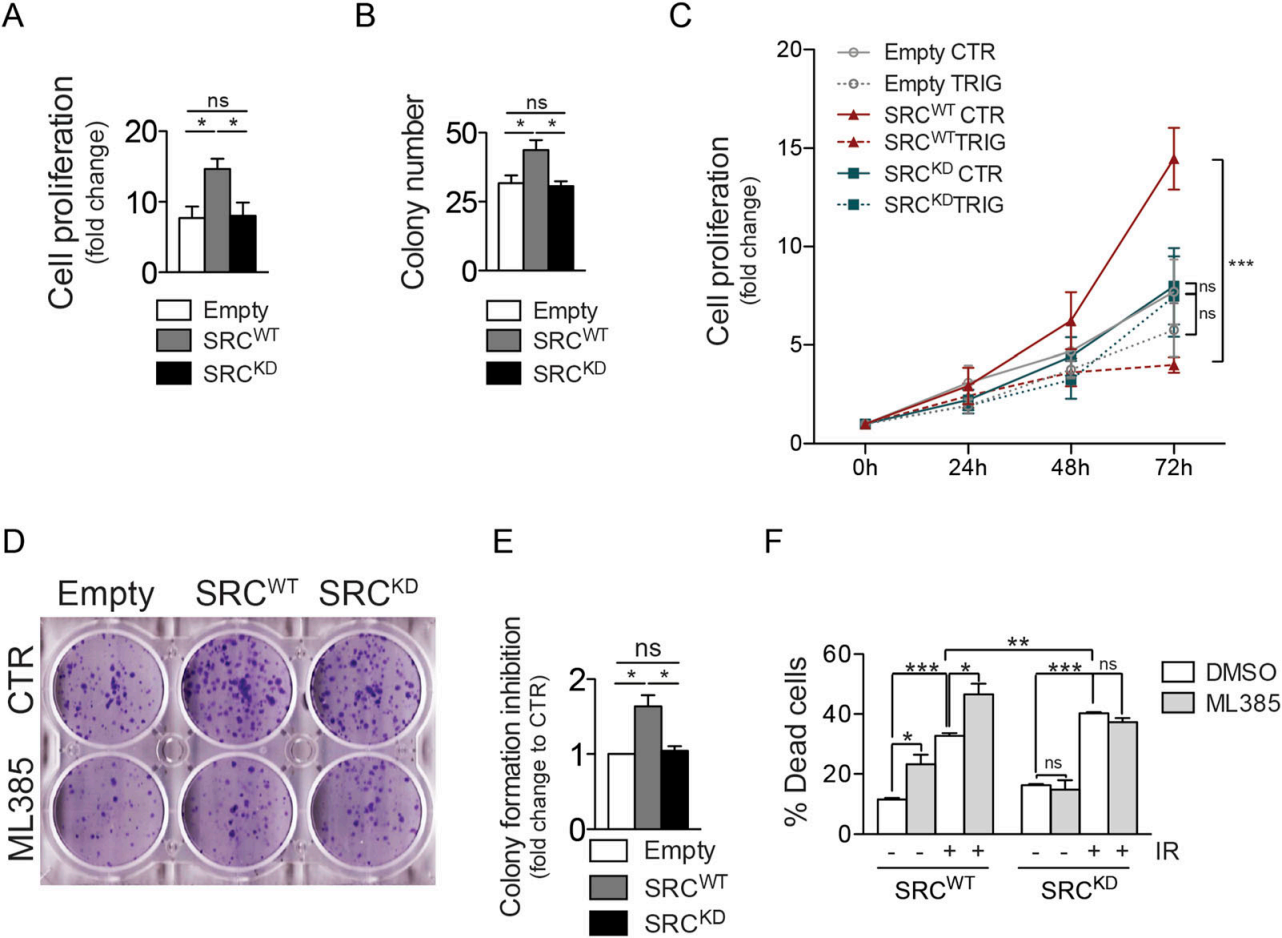
NRF2 inhibition affects cell proliferation and sensitivity to ionizing radiation in SRCWT cells. **(A, B)** Cell proliferation assay and (B) clonogenic assay performed in T98G cells stably overexpressing empty vector (empty), Src^WT^ or Src^KD^. **(C)** Cell proliferation assay performed in the same cells treated or not with trigonelline 5 μM for 0, 24, 48, and 72 h. **(D, E)** Clonogenic assay and (E) relative histogram of T98G cells stably overexpressing mepty vector (empty), Src^WT^ or Src^KD^ and treated or not with ML385 5 μM. **(F)** Histogram of the percentage of cell death from cytofluorimetric analysis in T98G cells stably overexpressing Src^WT^ or Src^KD^ 48 h from irradiation (10 Gy), upon pretreatment or not with ML385 5 μM. Results represent the mean of at least three independent experiments (±SEM). Statistical analyses: unpaired *t* test: (*P < 0.05; **P < 0.01; ***P < 0.001). Source data are available for this figure.

It has been shown that increased NRF2 protein levels represent a major trigger for cancer resistance to therapy (Awuah et al, 2022). NRF2 indeed is strongly involved in tumor growth (Rojo de la Vega et al, 2018), it can sustain and promote malignant transformation of GBM stem cells (Zhu et al, 2013) and it has been shown to be responsible for chemo and radiotherapy resistance in GBM cellular models (Singer et al, 2015; Rocha et al, 2016) shifting resistant cells towards mesenchymal phenotype (Singer et al, 2015). We therefore asked whether turning off NRF2 functionality in tumors that up-regulate Src activity may enhance the therapeutic response. Firstly, we showed that cell proliferation and clonogenic potential of Src^WT^ cells are significantly higher compared with Src^KD^ cells or cells overexpressing the empty vector (Fig 7A and B). In addition, we could show that NRF2 inhibition by trigonelline treatment significantly affected cell proliferation only in Src^WT^ cells (Fig 7C). In line with this, clonogenic assay experiments demonstrated that NRF2 inhibition via ML385 has a stronger effect on the clonogenic potential of Src^WT^ cells (Fig 7D and E).

To further strengthen NRF2 role downstream Src activity, we evaluated GBM cells sensitivity to ionizing radiation (IR), which represents the standard therapeutic approach for GBM patients. As expected, Src^KD^ cells were slightly but significantly more sensitive to IR than Src^WT^ cells (Fig 7F). Of note, NRF2 inhibition significantly affected cell viability in Src^WT^ and not in Src^KD^ cells and significantly increased sensitivity to IR-induced cell death only in Src^WT^ cells (Fig 7F).

### Src kinase activity drives NRF2-dependent resistance to ionizing radiation-induced ferroptosis

**Figure 8.**
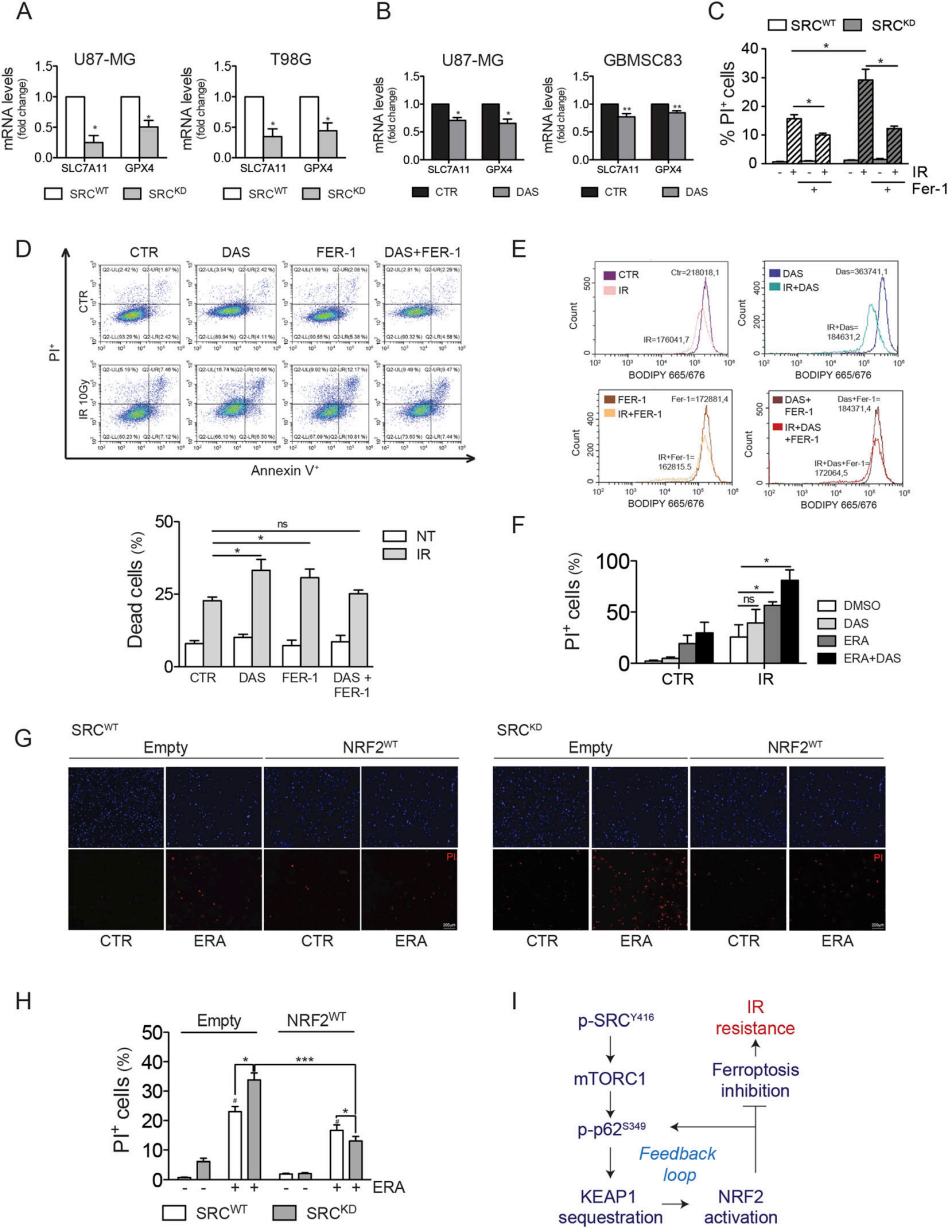
NRF2 inhibition sensitizes glioblastoma cells to ionizing radiation-induced ferroptotic cell death. **(A, B)** Real-time PCR of ferroptotic genes *slc7a11* and *gpx4* in (A) U87-MG and T98G stably expressing SRC^WT^ or SRC^KD^ and in (B) U87-MG and GBMSC83 cells treated with dasatinib (DAS) 10 nM and 1 μM, respectively (actin: housekeeping gene). **(C)** Histogram of propidium iodide-positive (PI^+^) T98G SRC^WT^ and SRC^KD^ cells after 48 h from irradiation (IR, 10 Gy), with or without Fer-1 5 μM treatment (SRC^KD^ cells: gray columns; IR: stripped bars). **(D)** Representative flow cytometry dot plot graphs and relative histogram of the percentage of dead cells upon AnnexinV-PI staining of GBMSC83 cells after 48 h irradiation (IR 10 Gy) with or without DAS 1 μM and/or Fer-1 5 μM (NT = untreated, white columns; IR = gray columns). **(E)** Cytofluorimetric analysis of lipid peroxidation upon BODIPY 665/676 staining in GBMSC83 cells after 48 h irradiation (10 Gy), with or without DAS 1 μM and/or Fer-1 5 μM. **(F)** Histogram of propidium iodide-positive (PI^+^) T98G cells after 48 h irradiation (10 Gy), with or without DAS 10 nM and/or erastin (ERA) 1 μM. **(G, H)** Representative images and (H) relative histogram of Propidium Iodide positive (PI^+^) T98G SRC^WT^ and SRC^KD^ cells transiently transfected with empty vector or NRF2^WT^, treated or not with erastin (ERA) 1 μM for 48 h. **(I)** Working model. Results represent the mean of three independent experiments (±SEM). **(A, B, C, D, G, I)** Statistical analyses: paired (A, B) and unpaired (C, D, G, I) *t* test: (*P < 0.05; **P < 0.01; ***P < 0.001). Source data are available for this figure.

**Figure S8.**
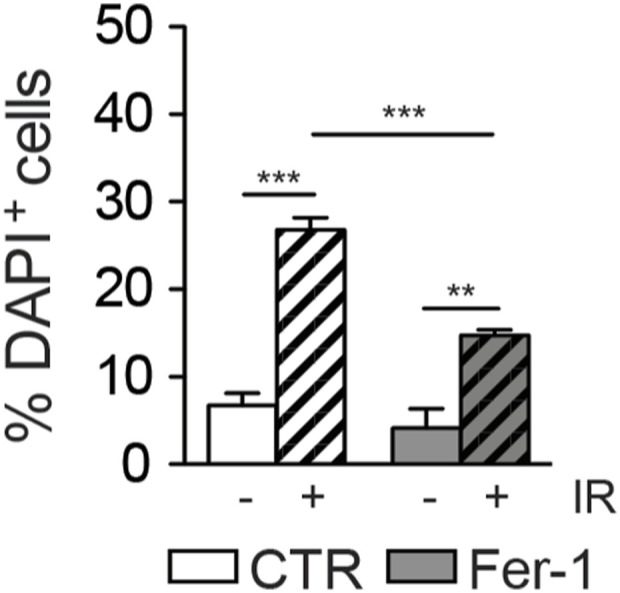
Histogram of DAPI^+^ T98G cells after 48 h from irradiation (IR, 10 Gy), with or without ferrostatin-1 (Fer-1) 5 μM pretreatment (Fer-1: gray columns; IR: stripped bars). Source data are available for this figure.

**Figure S9.**
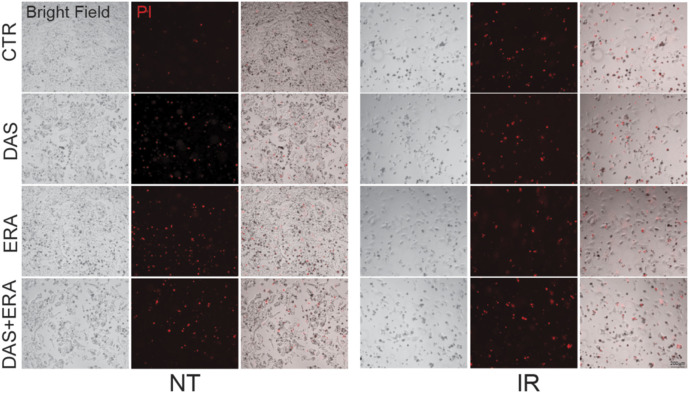
Representative Brightfield images and propidium iodide (PI^+^, red) staining in T98G cells after 48 h irradiation (10 Gy), with or without dasatinib 10 nM and/or erastin (ERA) 1 μM. Statistical analyses: paired *t* test: (*P < 0.05; **P < 0.01).

Recently, a novel role of NRF2 as inhibitor of ferroptosis, has been reported also in GBM (Fan et al, 2017; Dodson et al, 2019). Importantly, *slc7a11* and *gpx4*, two genes that play a key role in the modulation of ferroptosis, have been also shown to be NRF2 target genes (Wu et al, 2019). We therefore asked the question whether Src–NRF2 axis may also sustain the up-regulation of these genes, preventing ferroptosis. Genetic (Src^KD^ cells) and pharmacological (DAS) inhibition of Src kinase activity significantly decreased the expression of *slc7a11* and *gpx4* genes (Fig 8A and B). Given these data, we sought to investigate the significance of ferroptosis in our experimental models. After 48 h from irradiation, we observed a significant reduction in the percentage of cell death when ferroptosis was inhibited by ferrostatin-1 (Fer-1) (Fig S8), supporting the conclusion that IR-induced cell death occurs also via ferroptosis in our cells. The role of tyrosine kinases in the modulation of ferroptosis has not been clearly investigated yet. We asked whether the increased sensitivity to IR detected in Src^KD^ cells (Fig 7F) could be because of ferroptosis. Indeed, the inhibition of ferroptosis has a more dramatic effect on Src^KD^ cells compared with the Src^WT^ ones (Fig 8C). To strength the significance of our finding, we also performed experiments on patient-derived GBMSC83 neurospheres. We observed that DAS treatment sensitized GBMSC83 to IR. Importantly, co-treatment with DAS and Fer-1 failed to enhance the percentage of cell death (Fig 8D), thus suggesting that indeed Src inhibition sensitize cells to ferroptosis. In line with this, lipid peroxidation increased upon DAS and irradiation co-treatment (Fig 8E). Importantly, ferroptosis induction by erastin was strongly induced by the combined treatment with DAS and, more interestingly, this combination strongly sensitized cells to IR (Figs 8F and S9).

To further demonstrate that Src overexpression requires NRF2 to inhibit ferroptosis in GBM cells, we compared sensitivity with erastin in Src^WT^ and Src^KD^ cells transiently overexpressing NRF2^WT^, or an empty vector as a control. As shown, NRF2^WT^ overexpression significantly protects Src^KD^ cells from ferroptosis while having little and nonsignificant effect on Src^WT^ cells (Fig 8G and H).

Altogether, these data demonstrate that the Src–NRF2 axis sustains cancer cell resistance to ionizing radiation by preventing ferroptosis (Fig 8I).

## Discussion

Radiation and chemotherapy resistance is a major issue in cancer therapy as cancer cells activate several signaling pathways to counteract cell death induction. Ferroptosis inhibition is emerging as one of the major mechanisms responsible for IR resistance (Chen et al, 2021), although the molecular mechanisms that allow cancer cells to prevent ferroptosis have been only partially elucidated.

NRF2 transcription factor, a major controller of oxidative stress, has been shown to be aberrantly up-regulated in many tumors, including GBM (Rojo de la Vega et al, 2018). More importantly, its overexpression can drive cancer resistance to chemo and radiotherapy-induced apoptosis and ferroptosis, therefore representing one of the major triggers of poor prognosis in these tumors (Wu et al, 2019). Its aberrant activation relies often on NRF2 or KEAP1 gene mutation, both resulting in increased NRF2 protein levels (Kansanen et al, 2013). Nevertheless, the up-regulation of NRF2 has been also detected independently of these events pointing to alternative signaling molecules hyperactivated in cancer as possible NRF2 regulators. Recently, the bioinformatics analysis of gene expression data from the Broad–Novartis Cancer Cell Line Encyclopedia (CCLE) and TCGA identified GBM as one of the tumors where NRF2 is more often up-regulated (Pölönen et al, 2019). We confirmed this issue (Fig 1) and we therefore selected GBM cellular models as experimental systems to test the hypothesis that other signaling pathways often deregulated in GBM may contribute to increase NRF2 expression levels. Genomic and transcriptomic analyses uncovered RTKs signaling as one of the pathways that are more frequently deregulated in GBM (Cancer Genome Atlas Research Network, 2008). More than 60% of GBM display aberrant activation of EGFR or MET or HER2. Importantly, a common trait of the signaling cascade downstream RTKs is the constitutive activation of Src kinase (Du et al, 2009). Based on these pieces of evidence, we decided to test the hypothesis that Src activity may impact on NRF2 expression and signaling. Here, by using GBM cell lines, we identified for the first time a role for Src kinase activity as modulator of NRF2. Importantly, we confirmed that in our experimental systems both NRF2 protein expression and Src activity were up-regulated (Fig 1) and that both pharmacological and genetic inhibition of Src kinase activity significantly down-regulated NRF2 protein expression levels (Figs 1 and 2). Immunofluorescence and subcellular fractionation experiments showed that Src sustains NRF2 localization in the nucleus (Fig 2), thus promoting the expression of selected NRF2-target genes related to the oxidative stress response (Fig 3) and to ferroptosis (Fig 8). We also reported that Src caused the formation of p62 aggregates that colocalize with KEAP1. On the other hand, Src kinase activity inhibition resulted in the release of p62–KEAP1 complex and led to KEAP1 association with NRF2, which can be therefore ubiquitinated and degraded (Figs 4 and 5). Our findings are in agreement with previous reports demonstrating that NRF2 and p62 can compete for KEAP1 binding (Jain et al, 2010; Komatsu et al, 2010; Lau et al, 2010). In addition, p62 is a major target of NRF2 transcription factor pointing to a positive feedback loop mechanism (Jain et al, 2010). Interestingly, we could demonstrate that the constitutive activation of Src relies on mTORC1 signaling to promote this signaling pathway (Figs 6, S5, and S6). Our observations are consistent with recent studies that identified the aberrant activation of mTORC1 in GBM and in HCC cellular models as responsible for the up-regulation of NRF2 (Komatsu et al, 2010; Pölönen et al, 2019). We can speculate that the aberrant activation of Src in cancer cells constitutively activates NRF2 to survive to unfavorable conditions. Importantly, we also demonstrated that Src activity promotes the expression of p62, (Fig 3) that when overexpressed, may further sustain mTORC1 signaling and NRF2 activation (Duran et al, 2011).

We reported that NRF2 acts as a downstream effector for Src deregulation in cancer, supporting cell proliferation and cell resistance to radiotherapy (Fig 7). Importantly, Src-dependent NRF2 activation may contribute to inhibit ferroptosis and indeed its targeting may increase sensitivity to ferroptosis, enhancing their efficacy in combination with IR (Fig 8).

The link between tyrosine kinases, NRF2 signaling, and ferroptosis is still under-investigated. As NRF2 transcription factor controls several cellular responses, it will be interesting to perform transcriptomic analyses to further elucidate the panel of NRF2 target genes that may be modulated by Src kinase activity.

Our work suggests that the concomitant increased activity of Src and NRF2 may identify those tumors that may benefit from targeting this novel signaling axis. Of note, among GBM tumors, the mesenchymal subtype characterized by increased NRF2 expression (Pölönen et al, 2019) has also been shown to be more sensitive to DAS treatment (Alhalabi et al, 2022). Because the constitutive activity of Src is a major feature of cancer, it will be interesting to extend the investigation to different tumors. Of note, because of the redundancy of tyrosine kinase signaling and being Src aberrantly induced downstream the activation of RTKs, we cannot exclude a role of other tyrosine kinases and we can speculate that RTKs may provide a general mechanism to impinge on NRF2 signaling independently of specific NRF2 or KEAP1 mutations. In summary, we provide the first evidence for a new connection between the aberrant activation of tyrosine kinases and the constitutive activation of NRF2 signaling, and we identify an unexpected role for tyrosine phosphorylation signaling in the modulation of ferroptosis.

## Materials and Methods

### Cell culture

U87MG and T98G (originally obtained by ATCC) were cultured in DMEM supplemented with 10% FBS, 100 U/ml penicillin, and 100 mg/ml streptomycin (Sigma-Alrich). GBMSC83 cells, a well-characterized mesenchymal GBM cellular model, were cultured as neurospheres in non-adherent conditions in DMEM/F12 supplemented by B27 Supplement (50x), EGF (20 ng/ml), and hβFGF (10 ng/ml) as previously described (Mao et al, 2013; Minata et al, 2019). All GBM cells were maintained in a humidified 5% CO, 37°C incubator and were routinely tested negative for mycoplasma contamination.

Stable cell lines, overexpressing Src *wild-type* or catalytically inactive mutant *kinase dead* in p-MX-psCESAR vector, were generated by retroviral infection followed by cell sorting for GFP^+^ cells. For transient transfection experiments, cells were seeded the day before and transfected using polyethylenimine (PEI) (Tebu-Bio), following the manufacturer’s instructions. Src constructs pSGT-Src-Y527F and pSGT-K295M and empty pSGT vector were previously described (Cursi et al, 2006). pCDNA3.1-FLAG-NRF2^WT^ was obtained from Addgene.

### Antibodies and other reagents

Primary antibodies used are as follows: anti-NRF2 (D1Z9C) (Cell Signaling Technology), anti-p62 (SQSTM1) (PM045; MBL International), anti-phospho-p62 (SQSTM1) (Ser349) (PM074; MBL International), anti-vinculin (E1E9V) (Cell Signaling Technology), anti-lamin (E-1) (sc-376248; Santa Cruz Biotechnology), anti-GAPDH (D16H11) (Cell Signaling Technology); anti-Src (2108S) (Cell Signaling Technology), anti-phospho-Src (Tyr416) (2101S) (Cell Signaling Technology), anti-KEAP1 (G-2) (sc-365626; Santa Cruz Biotechnology), anti-heme oxygenase 1 (A-3) (sc-136960; Santa Cruz Biotechnology), anti-NQO1 (A-180) (sc-32793; Santa Cruz Biotechnology), anti-p70S6K (Cell Signaling Technology), anti-phospho-p70S6K (Thr389) (Cell Signaling Technology), anti-ERK 1/2 (137F5) (Cell Signaling Technology), anti-phospho-ERK 1/2 (Thr202/Tyr204) (D13.14.4E) (Cell Signaling Technology), anti-ubiquitin (P4D1; Santa Cruz Biotechnology).

Dasatinib, trigonelline, rapamycin, ferrostatin-1, erastin, and cycloheximide were purchased from Sigma-Aldrich; PP2 (sc-202769) from Santa Cruz Biotechnology; ML385 from Selleck Chemicals.

### Fluorescence microscopy

Cells were seeded on coverslips and grown at 37°C and 5% CO_2_. After treatments, cells were washed with 1x PBS, fixed in 4% PFA for 15 min at RT, permeabilized with PBS/Triton X-100 0.3% solution for 10 min, blocked with BSA 3% solution in PBS for 1 h, and incubated overnight with primary antibodies (NRF2, 1:50; KEAP1, 1:50; p62: 1:1,000) in a humid chamber at 4°C. Secondary antibodies (1:500; Thermo Fisher Scientific) were applied for 1 h at RT, and nuclei were stained with Hoechst 33342 (Thermo Fisher Scientific) for 15 min. Images were acquired by fluorescence microscopy (ZEISS) and processed with Fiji version 2.3.

Confocal microscopy experiments were performed by using LSM800 microscope (ZEISS) equipped with a 63x oil objective and with ZEN blue imaging software. Staining intensities were analyzed by using Fiji software (ImageJ) to obtain the nuclear/cytoplasmatic ratio. For colocalization studies, at least three slices were taken at a Z-stack distance of 0.3 μm. After acquisition, images were imported into Fiji for analysis and quantification of colocalizing particles between two channels by using the open-source plugin ComDet 0.5.2. Colocalization parameters were as follows: maximum distance between the center of two particles ≤ 4 pixels; particle size ≥ 3; intensity threshold = 5.

### Protein extracts, immunoprecipitation, nuclei/cytoplasm fractionation, and immunoblotting analyses

Total cell extracts were prepared in Buffer A (10 mM Hepes [pH 7.9], 10 mM KCl, 1.5 mM MgCl_2_, 0.5 mM DTT, 0,1% NP-40, 5 mM EDTA, 5 mM EGTA, 1 mM phenylmethylsulfonyl fluoride, 25 mM NaF, 1 mM sodium orthovanadate, 10 mg/ml TPCK, 5 mg/ml TLCK, 1 mg/ml leupeptin, 10 mg/ml soybean trypsin inhibitor, 1 mg/ml aprotinin). Lysates were incubated for 20 min on ice, sonicated, and centrifugated 12,000*g* at 4°C for 30 min.

For nuclei/cytoplasm cell fractionation, cells were lysate in the same Buffer A, without adding 0.1% NP-40. After incubation for 20 min on ice, NP-40 (0.1% final concentration) was added, and nuclei were harvested by centrifugation at 12,000*g* at 4°C for 30 s. The cytoplasmic fraction was recovered, and nuclear proteins were extracted from the pellet in Buffer A, completed with 0,05% NP-40 for 30 min on ice followed by sonication and centrifugation at 12,000*g* at 4°C for 30 s. For immunoprecipitation experiments, total cell extracts were incubated with the primary antibody for 3 h on a rotating wheel at 4°C, followed by 45 min incubation with Protein G beads (Invitrogen). The complex was washed four times in ice-cold PBS, denatured for 5 min at 95°C. For immunoblotting, 20–50 mg of proteins were separated by SDS–PAGE, blotted onto nitrocellulose membrane, and detected with specific antibodies.

### Cell death analysis

Cell death was evaluated 48 h after ionizing radiation (10 Gy) by using a CytoFLEX S (Beckman Coulter) instrument. 1 × 10^6^ cells were collected, centrifuged at 4°C for 5 min at 300*g*, and double-stained by using Annexin V-APC-propidium iodide (PI) kit, according to the manufacturer’s instructions (eBioscienceTM Annexin V Apoptosis Detection Kits; Thermo Fisher Scientific). For GFP^+^ cells, PI staining was replaced with DAPI. Unstained samples were used as control. Quality control was evaluated using CytoFLEX Daily QC Fluorospheres (Beckman Coulter). FCS files were analyzed using CytExpert version 2.2 software (Beckman Coulter). Dead cells (Annexin V+/PI+ cells) were graphed as fold change to control conditions.

### Proliferation assay

Cells were seeded in 12-well culture plate (1 × 10^5^ cells/ml) and incubated at 37°C, 5% CO_2_. After 24 h, cells were treated with trigonelline 5 mM. Cells were detached and counted 24, 48, and 72 h after treatment. Data were expressed as mean and SD. The assays were repeated four times.

### Clonogenic survival assay

Cells were plated in six-well culture plate (1,000 cells/well) and incubated at 37°C, 5% CO_2_ for colony formation. After 10–15 d, colonies were fixed and stained with a solution with 10% (vol/vol) methanol and 0.5% cristal violet for 20 min for colony visualization. The stained colonies (>50 cells) were counted. Data were expressed as mean and SD. The assays were repeated four times.

### RT–qPCR

Cells were homogenized in TRI Reagent (Thermo Fisher Scientific), and RNA was extracted in accordance with the manufacturer’s protocol. 1 mg of total RNA was retrotranscribed in cDNA using the SensiFAST cDNA Synthesis KIT (Bioline). Specific sets of primer pairs were designed and tested with primerBLAST (NCBI, see list below). RT–qPCR was performed using the SensiFAST Syber Low-ROX kit (Bioline) QuantStudio 3 RT–qPCR (Applied Biosystems). Data were analyzed by using the second-derivative maximum method. The fold changes in mRNA levels were determined relative to a control after normalizing to the internal standard, actin. Primers used are listed below:

**Table:** 

Gene	Forward primer	Reverse primer
ACTIN	5′-GGCCGAGGACTTTGATTGCA-3′	5′-GGGACTTCCTGTAACAACGCA-3′
HMOX-1	5′-CACAGCCCGACAGCATGCCC-3′	5′-GCCTTCTCTGGACACCTGACCCT-3′
NQO1	5′-GGTTTGGAGTCCCTGCCATT-3′	5′-CCTTCTTACTCCGGAAGGGTC-3′
GCLC	5′-CGCACAGCGAGGAGCTTCGG-3′	5′-CTCCACTGCATGGGACATGGTGC-3′
SQSTM1	5′-GGGAAAGGGCTTGCACCGGG-3′	5′CTGGCCACCCGAAGTGTCCG-3′
SLC7A11	5′TCCTGCTTTGGCTCCATGAACG-3′	5′-AGAGGAGTGTGCTTGCGGACAT-3′
GPX4	5′-GCCTTCCCGTGTAACCAGT-3′	5′-GCGAACTCTTTGATCTCTTCGT-3′

### Lipid peroxidation measurement

Membrane lipid peroxidation was evaluated 48 h after ionizing radiation (10 Gy), in the presence or not of DAS and ferrostatin-1. Cells were incubated for 30 min at 37°C with 5 mM of BODIPY665/676 probe (Thermo Fisher Scientific) dissolved in PBS, washed twice in PBS, and analyzed by using a CytoFLEX S (Beckman Coulter, Milan, Italy) instrument. An unstained sample was used as negative control. FCS files were analyzed using CytExpert version 2.2 software (Beckman Coulter).

### Publicly available datasets

The expression profile of GBM patients was downloaded from TCGA Data Portal (https://tcga-data.nci.nih.gov) using the recommended GDC data transfer tool. The processed data (level 3) were used. To date, there are 163 patients with unrestricted-for-publication RNA-seq(V2) data. The expression data of normal brain tissues were downloaded from GTEx portal (V8) (https://gtexportal.org/home/) where a total of 3,869 samples belonging to 13 different brain regions are available.

### Pathway variation analysis

Pathway enrichment score was calculated using the Gene Set Variation Analysis tool (Hänzelmann et al, 2013). A matrix containing pathway enrichment scores for each gene set and sample was obtained using the TCGA and GTEx gene expression matrices and the MSigDB Halmark gene set collection (Subramanian et al, 2005). Differential pathway activity between GBM and a normal brain cortex was calculated using Deseq2 (Love et al, 2014) and pathways with *P*-value < 0.05 and |log_2_ FC| > 0.3 were selected as significant.

### Statistical analyses

All experiments were replicated at least three times (biological replicates) and data were presented as mean ± SD or ± SEM, as indicated in the figure legends. The significance of the differences between populations of data was assessed according to the two-tailed *t* test (independent samples) with level of significance of at least *P* ≤ 0.05. For immunofluorescence analysis, unpaired *t* test was used for data showing a normal distribution and Mann–Whitney test in other situations. For multiple comparisons, we used ANOVA test followed by Kruskal–Wallis test. All the statistical analyses were performed using GraphPad Prism software.

## Supplementary Material

Reviewer comments
