# Supplementary figures and images for: NRF2 connects Src tyrosine kinase to ferroptosis resistance in glioblastoma
Source: Life Sci Alliance. 2023 Oct 25;7(1):e202302205. doi: 10.26508/lsa.202302205 (PMC10599979; doi:10.26508/lsa.202302205)

Original Files - Fig 1D

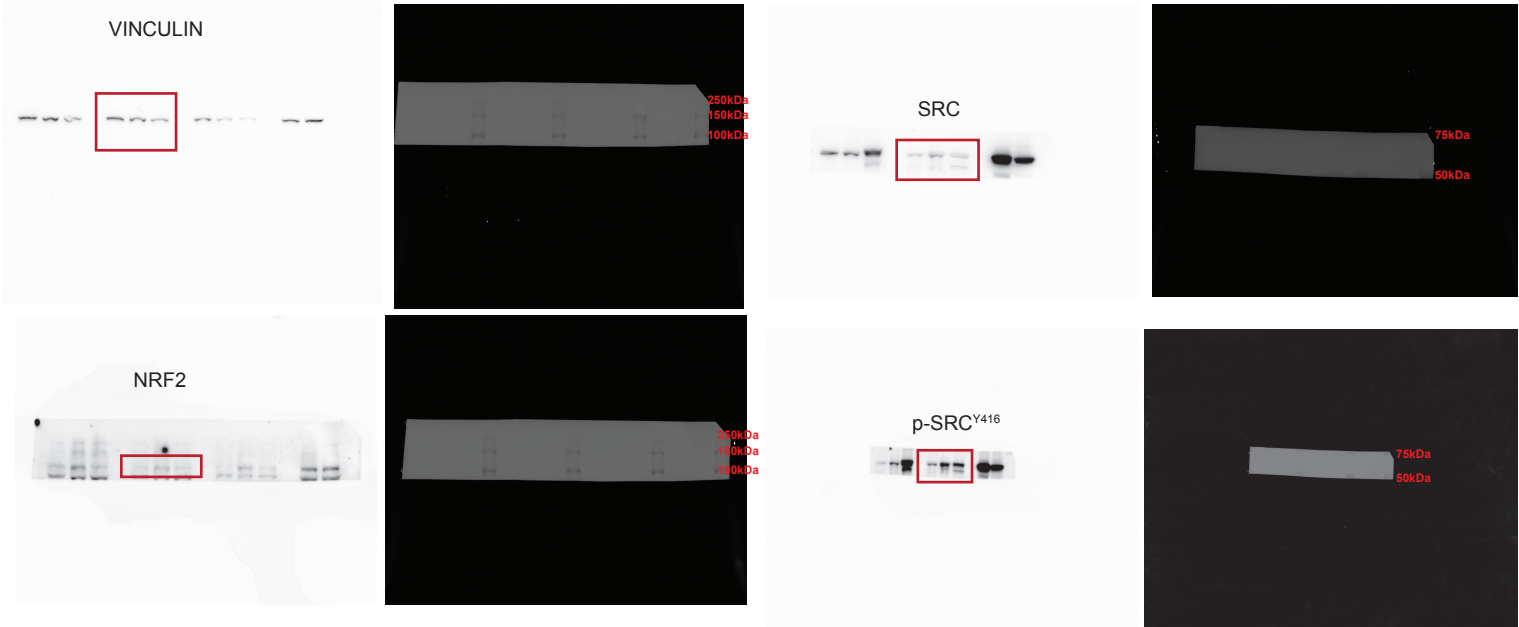

Supplement: Supplementary file 1 [file LSA-2023-02205_SdataF1.1.pdf]

Fig S1

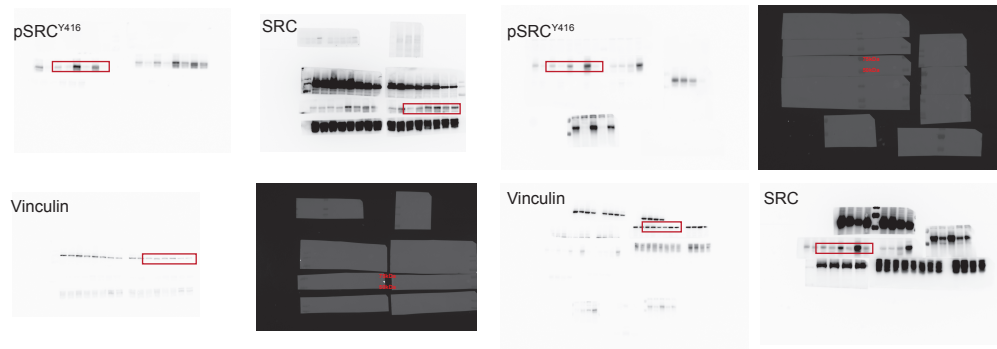

Fig S2

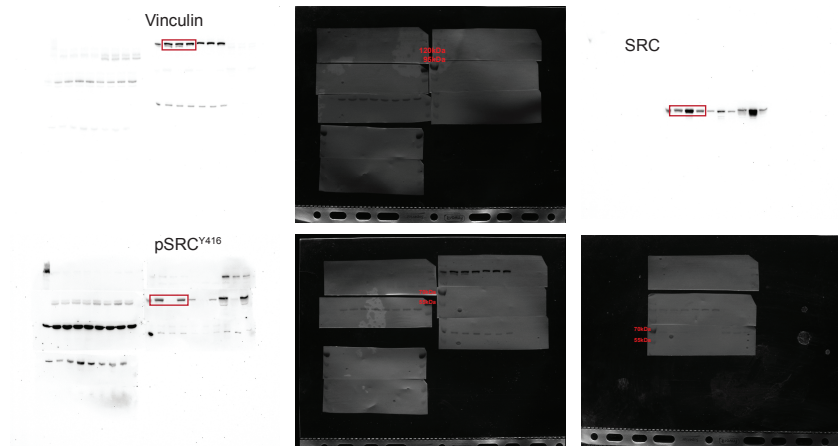

Fig S3

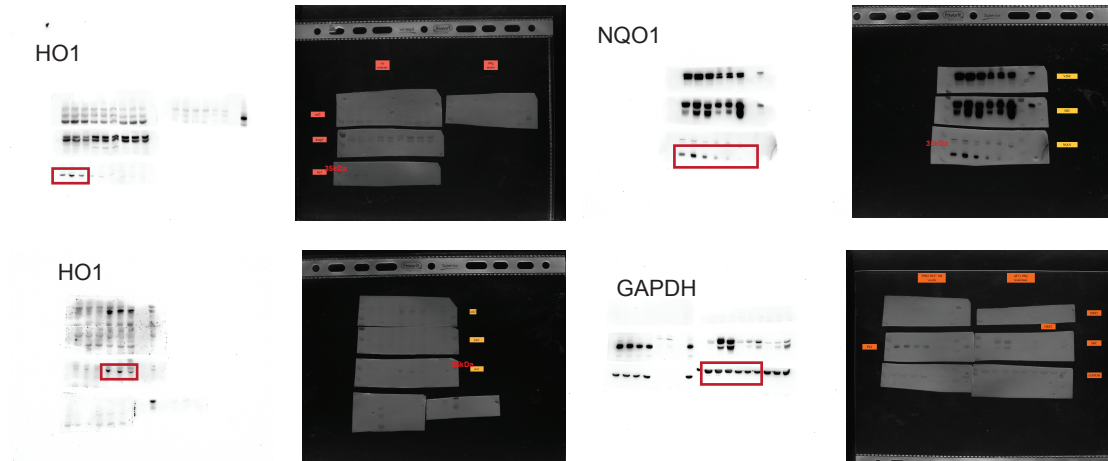

Fig S4

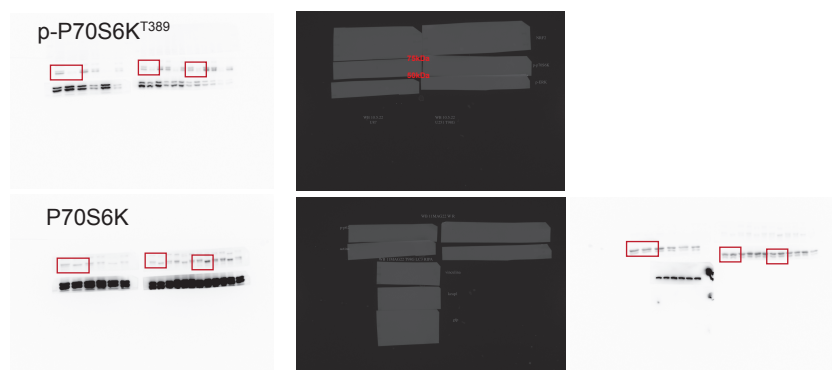

Supplement: Supplementary file 3 [file LSA-2023-02205_SdataFS1_FS2.2_FS3.1_FS4.pdf]

Original Files - Fig 2A + S2

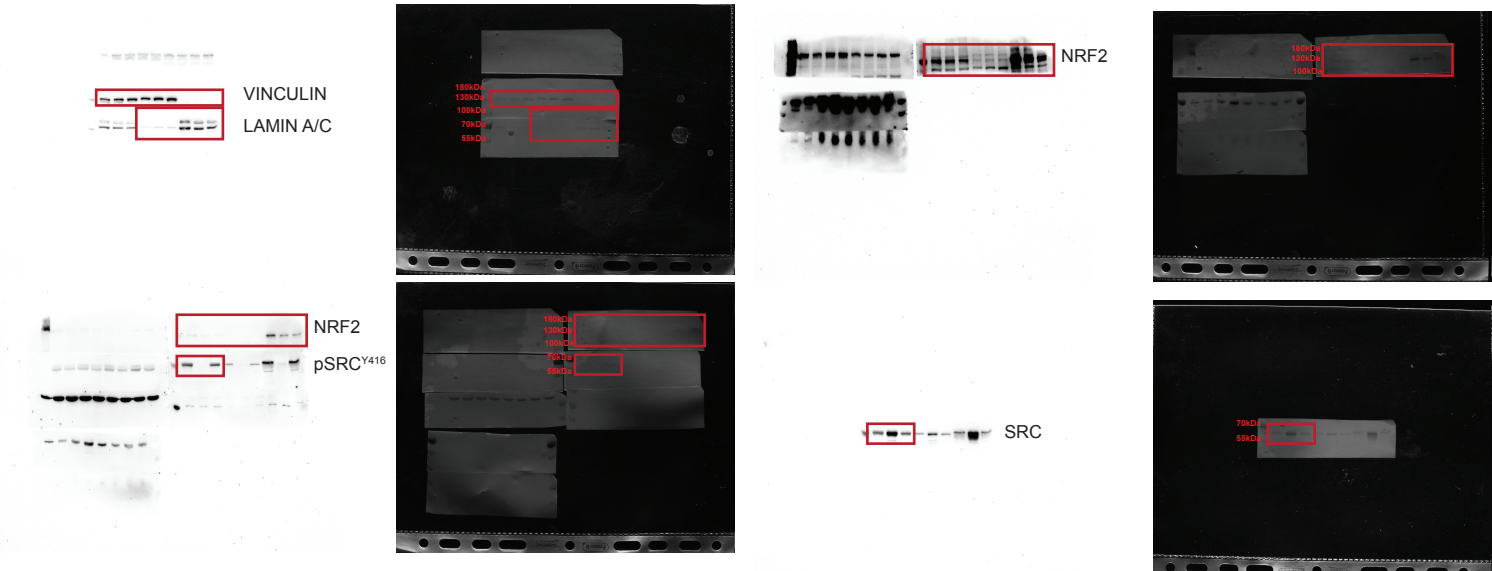

Original Files - Fig 2C

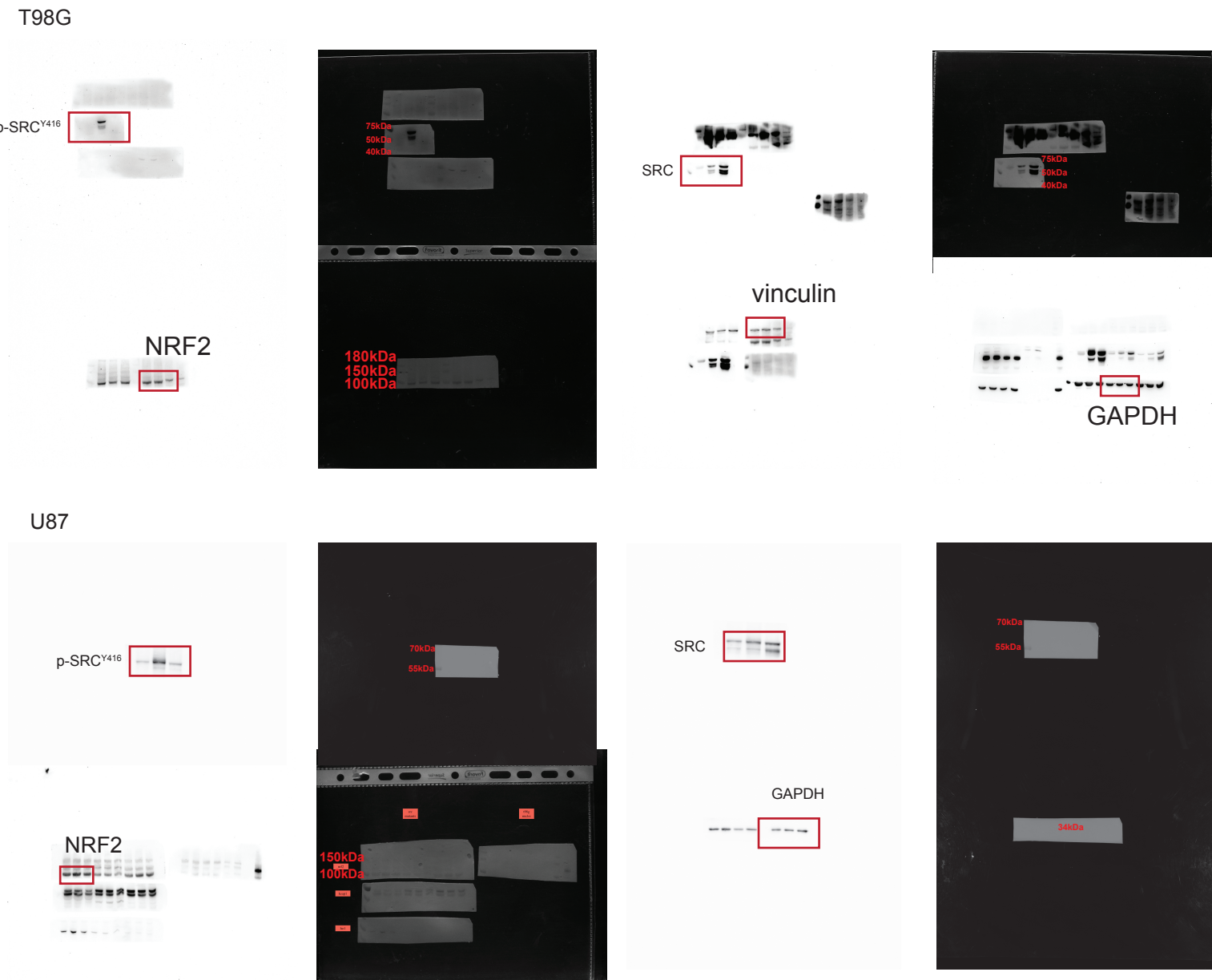

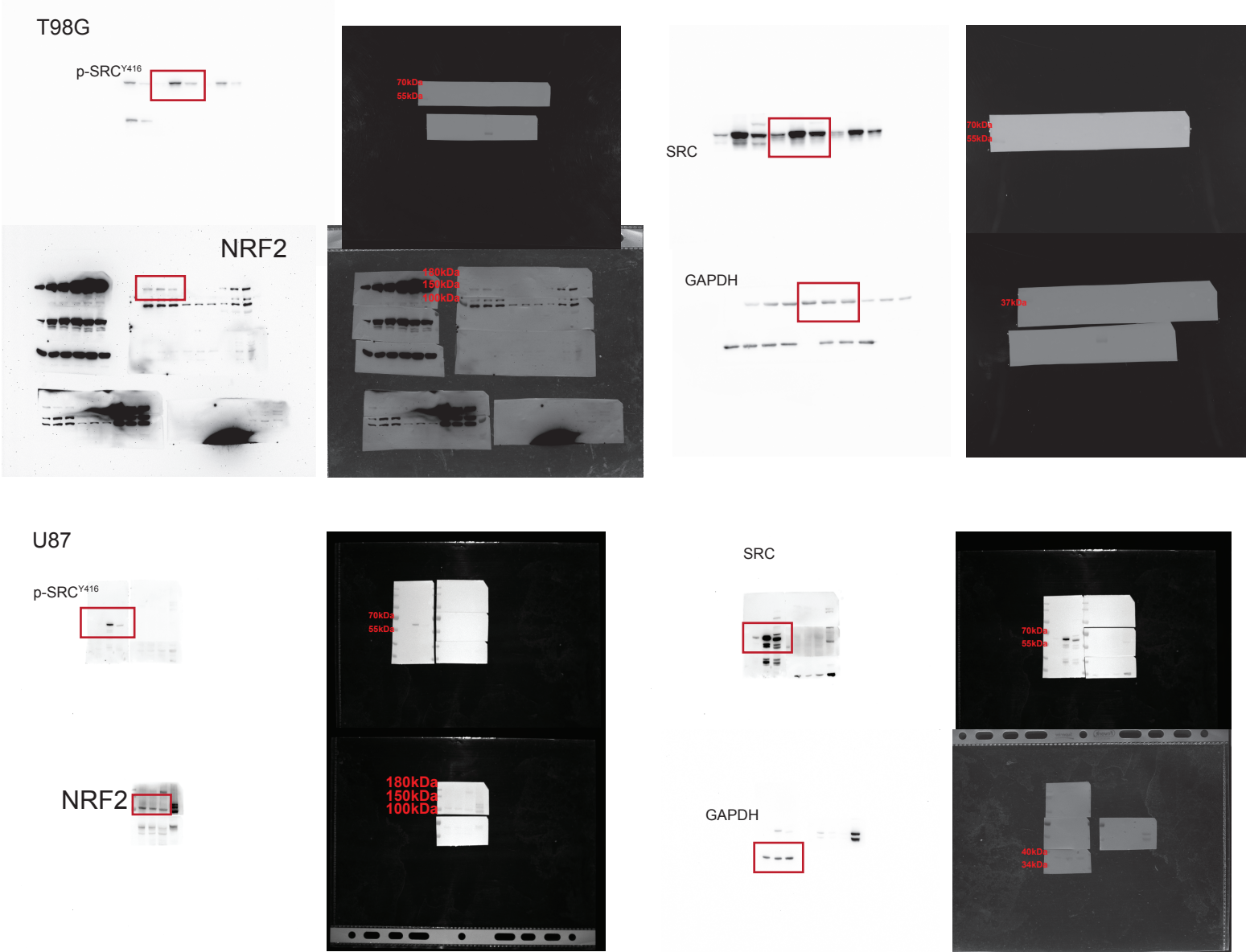

Supplement: Supplementary file 4 [file LSA-2023-02205_SdataF2.1_FS2.1.pdf]

Original Files - 3B

U87

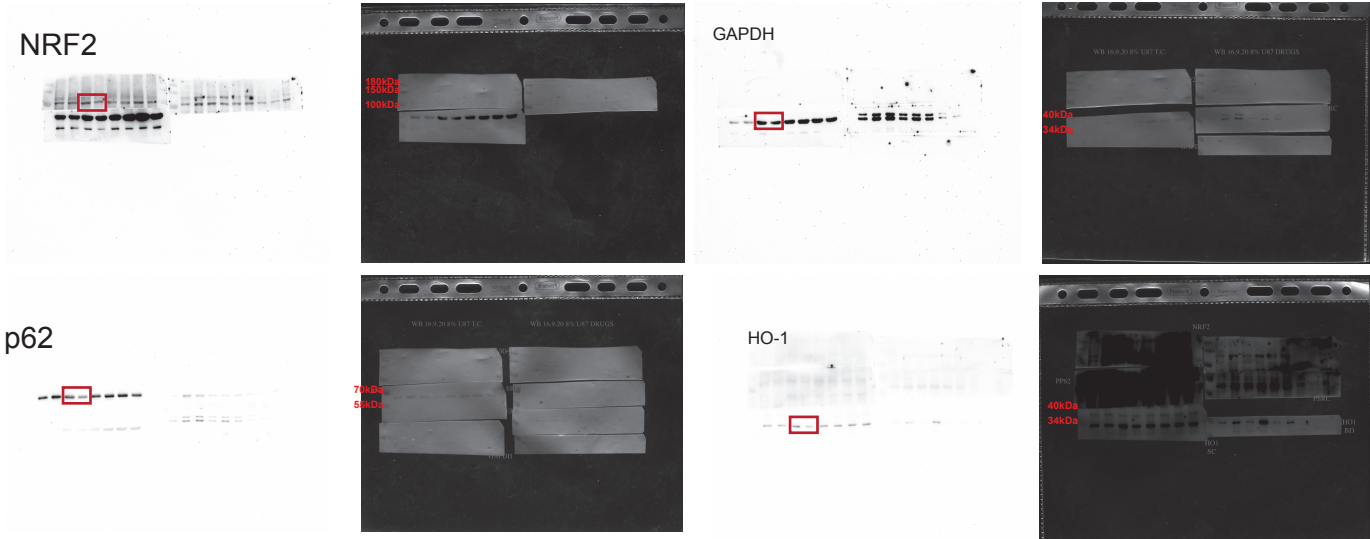

GBMSC83

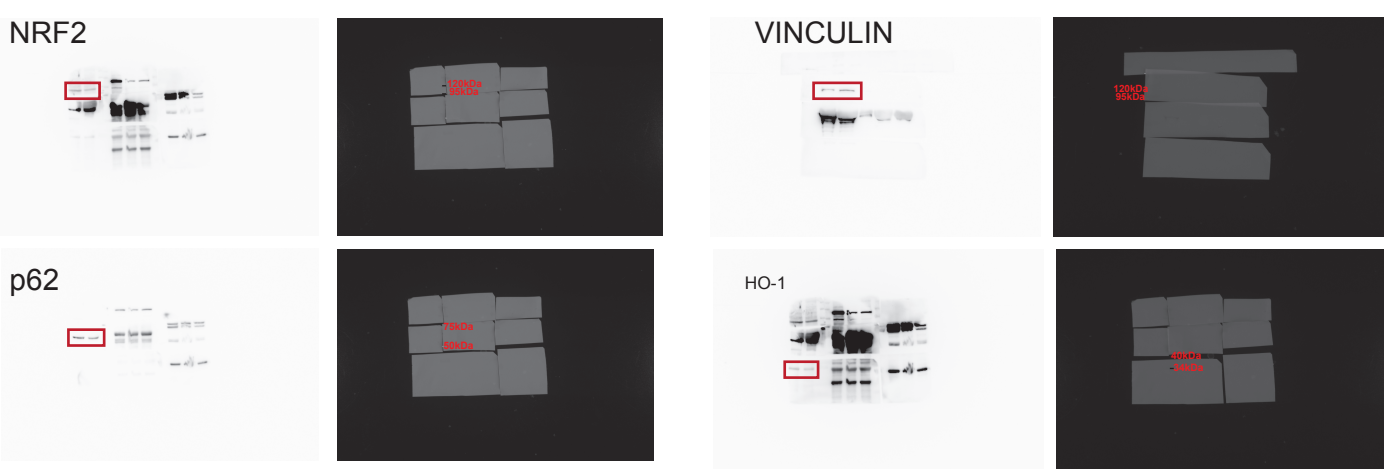

Original Files - 3D

U87

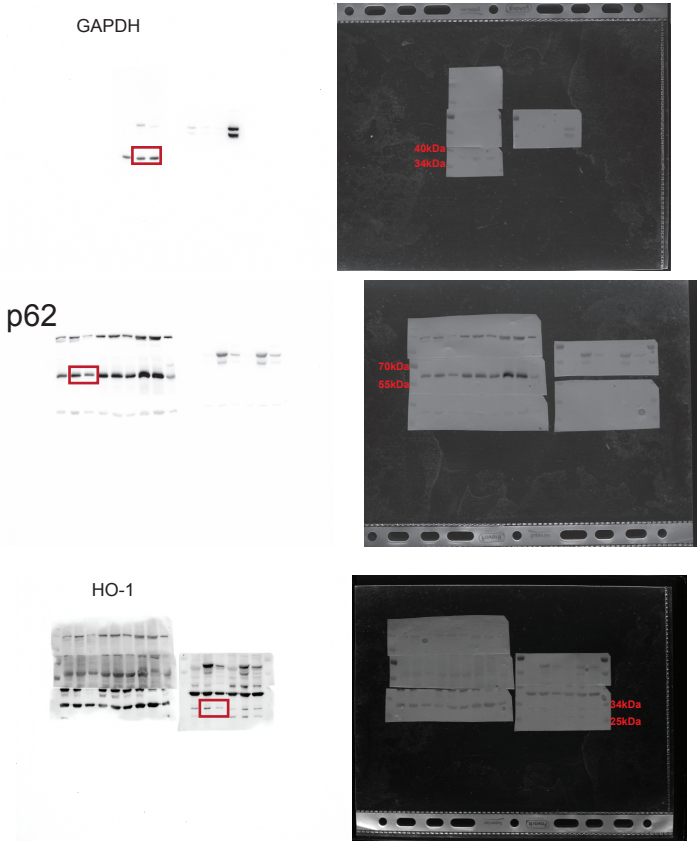

T98G

Supplement: Supplementary file 6 [file LSA-2023-02205_SdataF3.1.pdf]

Original Files - 4C

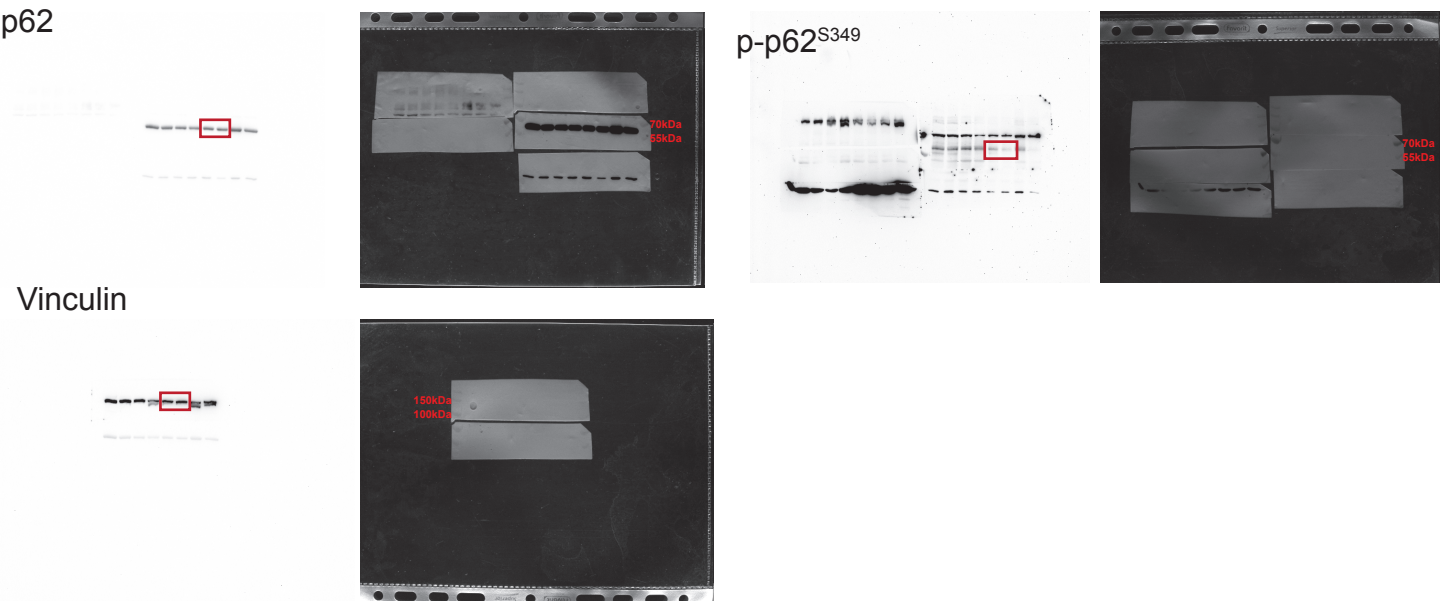

Original Files - 4F

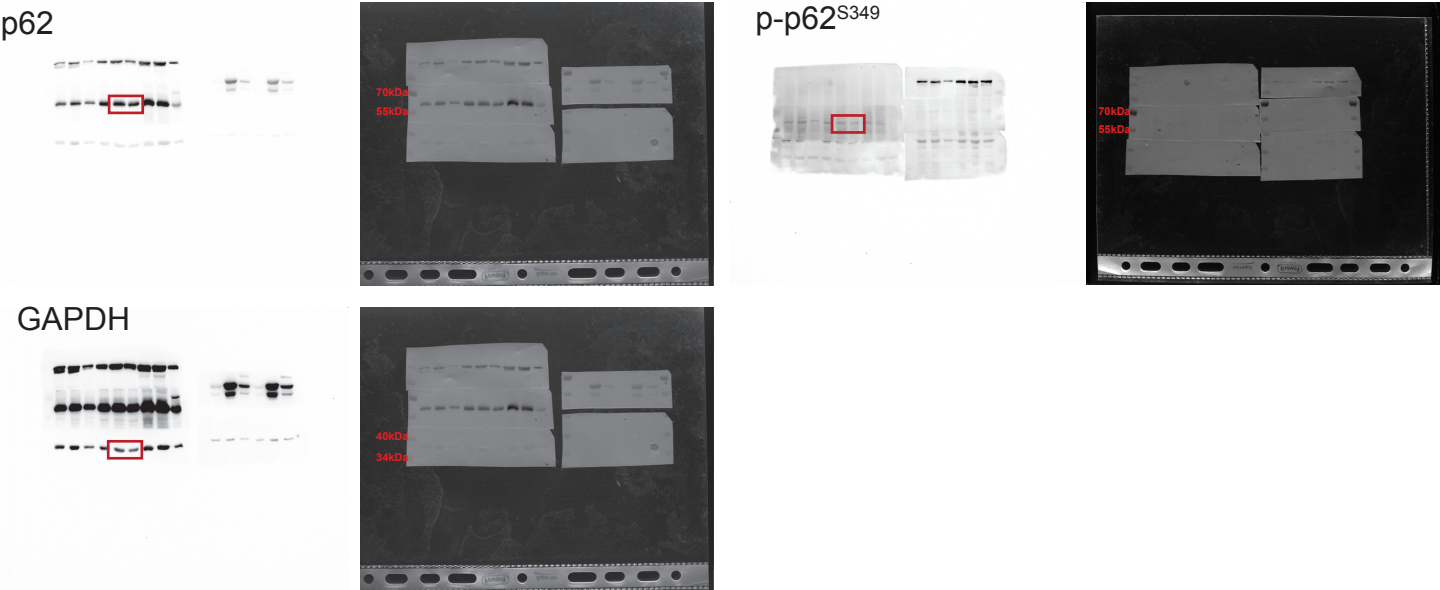

Supplement: Supplementary file 9 [file LSA-2023-02205_SdataF4.1.pdf]

Original Files - 5B

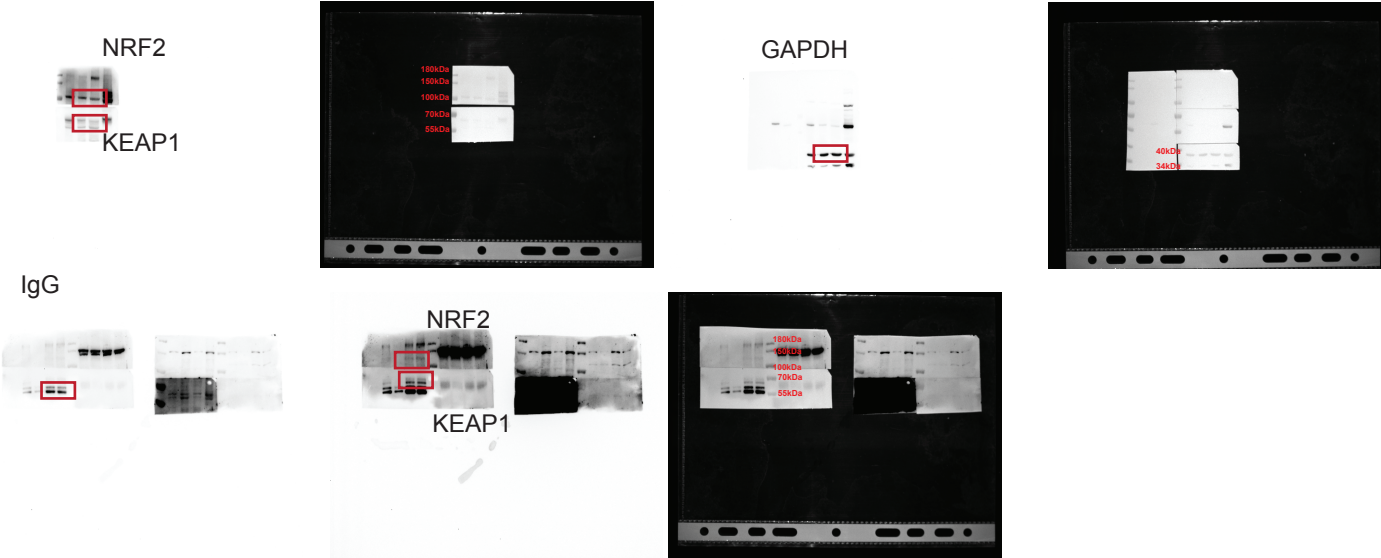

Original Files - 5C

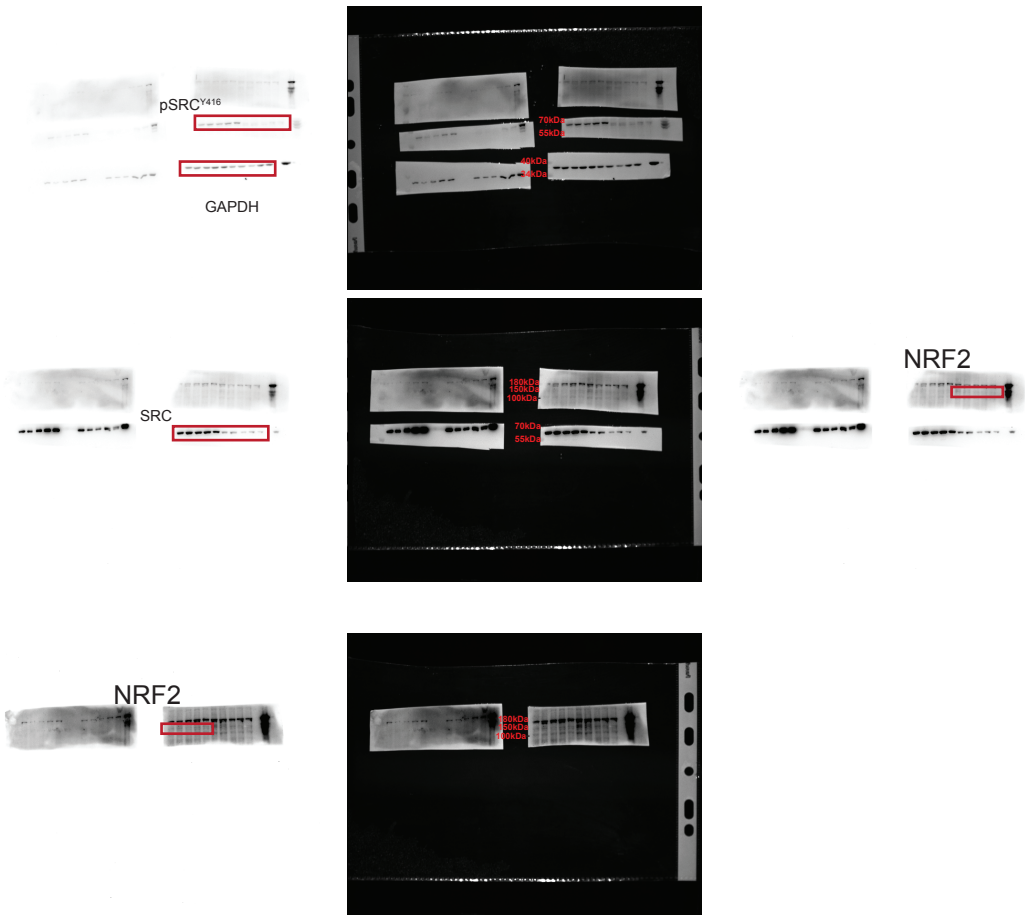

Supplement: Supplementary file 11 [file LSA-2023-02205_SdataF5.1.pdf]

Original Files - 6A

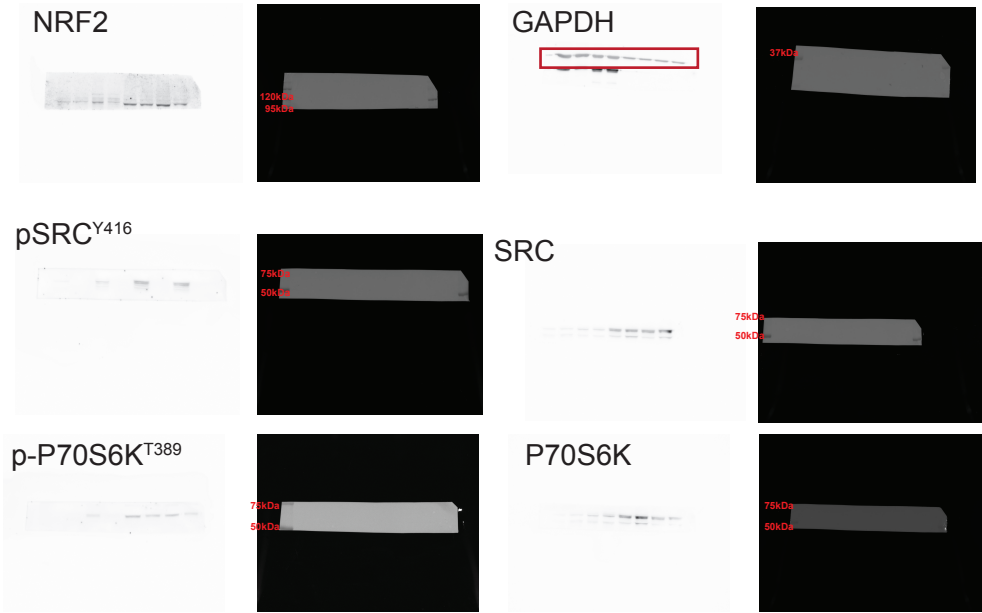

Original Files - 6B

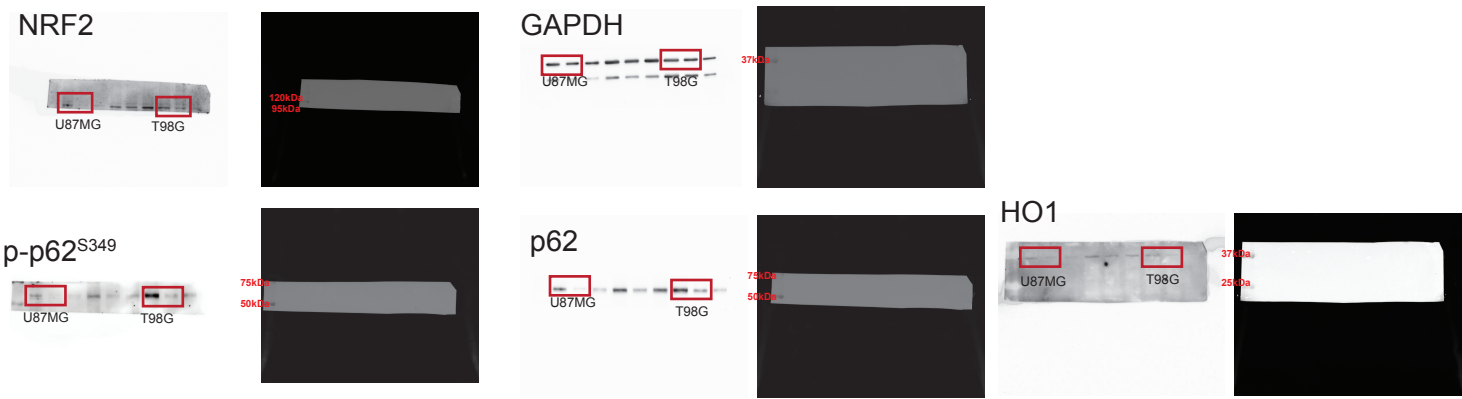

Supplement: Supplementary file 13 [file LSA-2023-02205_SdataF6.1.pdf]
